# Chromosome-level genome assembly of the Vermilion Snapper (*Rhomboplites aurorubens*)

**DOI:** 10.1038/s41597-025-05573-w

**Published:** 2025-07-23

**Authors:** A. Roa-Varón, P. Moulos, S. Harter, A. David, A. M. Quattrini, S. Herrera

**Affiliations:** 1 Department of Invertebrate Zoology, National Museum of Natural History, Smithsonian Institution, Washington, DC USA; 2 Institute for Fundamental Biomedical Research, Biomedical Sciences Research Center, Alexander Fleming, Fleming 34, 16672 Vari, Greece; 3 National Oceanographic and Atmospheric Administration, Southeast Fisheries Science Center, Panama City, FL USA; 4 Department of Biological Sciences, Lehigh University, Bethlehem, PA USA; 5 Lehigh Oceans Research Center, Lehigh University, Bethlehem, PA USA

**Keywords:** Genome, Marine biology

## Abstract

Vermilion Snapper (*Rhomboplites aurorubens*, Lutjanidae) inhabits deep waters (20–300 m) from North America to Brazil and supports significant commercial and recreational fisheries. Despite its economic importance, the understanding of its basic biology remains limited. Classified as Vulnerable on the Red List due to overfishing, populations have declined by over 30% in recent generations. We assembled and annotated the first chromosome-scale genome of this species by combining PacBio long reads, Illumina short reads, and Hi-C data. The resulting assembly is 987.5 Mbp, with a scaffold N50 size of 41.3 Mbp, and includes 135 contigs clustered and ordered onto 24 chromosomes with 34,496 predicted genes. The high-quality assembly and annotation contained about 98% complete and single-copy BUSCO genes. It is the most complete, chromosome-level genome assembly of an Atlantic snapper to date. The genome assembly and supporting data are valuable tools for ecological and comparative genomics studies of snappers and other valuable commercial species within the family.

## Background & Summary

**Fig. 1:**
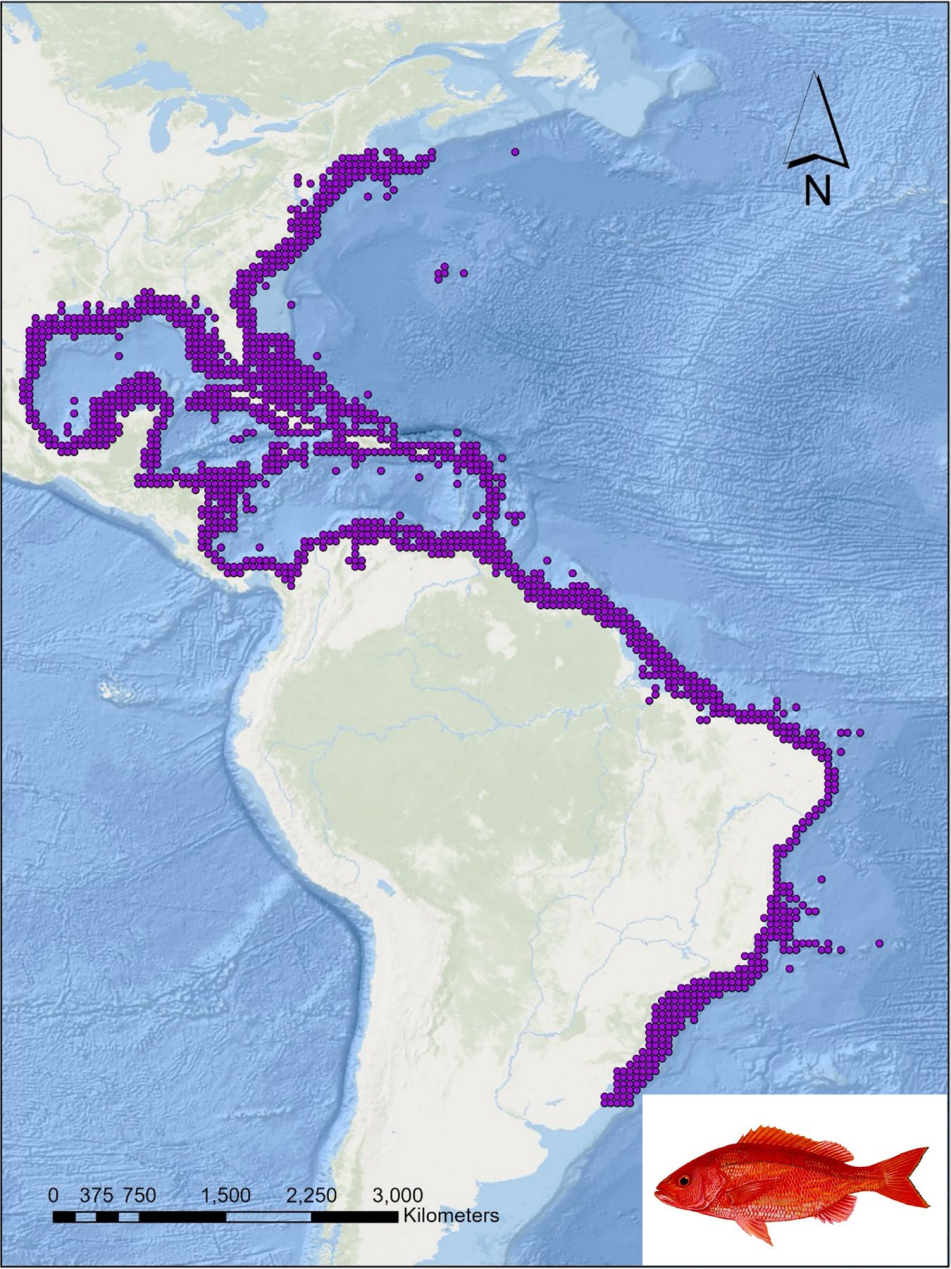
*Rhomboplites aurorubens* distribution. Data retrieved from AquaMaps (Kaschener *et al*., 2019).

Snappers (family Lutjanidae) are found worldwide in tropical and warm-temperate seas, ranging from the shoreline to continental slopes, and down to depths of 550 m (1,800 ft). The family comprises approximately 113 species, including 17 genera. Lutjanids are often associated with coral and rocky reefs^1^ throughout tropical seas, particularly the Indo-Pacific. *Rhomboplites aurorubens* (Vermilion Snapper) within the subfamily Lutjaninae occurs between 20 and 300 m depth in the western North Atlantic from North Carolina, United States to southeastern Brazil, including southern United States, Bermuda, and the Caribbean Sea^2,3^ (Fig. 1, Supplementary Table S1). Juveniles are found in shallow waters, while adults are found at deeper depths^4^. The species is associated with rocky bottoms, gravel, or sandy substrates near the edge of the continental shelves^4^ and feeds on pelagic and benthic organisms^1^, including fish and invertebrates. Vermilion snappers reach sexual maturity between 1 and 2 years old and have a lifespan exceeding 15 years^3^.

Vermilion Snapper is a target species for commercial, artisanal, and recreational fisheries that face significant fishing pressure. They are caught using hook and line, trawling, and traps. This species is listed as Vulnerable on the Red List of Threatened Species (IUCN)^5^ due to overfishing occurring in many areas of its range, resulting in an overall population decline of up to 30%^3^. Additionally, the mislabeling of Vermilion Snapper as wild-caught Red Snapper (*Lutjanus campechanus*) is widespread^6^ and could lead to the overexploitation of at-risk fish populations if fishing pressure increases^5^.

Genome assemblies are powerful tools in fishery assessments, providing comprehensive references for studying genetic diversity, population structure, and adaptive traits. In fisheries, this is critical for understanding species at a genetic level, especially for non-model or poorly studied fish species. Reference genome assemblies improve our ability to gain a deeper understanding of fish populations, supporting evidence-based management decisions to ensure long-term sustainability^7–9^. For the family Lutjanidae, the only available genome assembly is from a western Atlantic species, the Red Snapper, *Lutjanus campechanu*s (GCA_027495735.1); however, this genome is noncontiguous (67,254 scaffolds) and unannotated^10^. The other two genome assemblies available for the genus *Lutjanus* are the Indo-West Pacific snappers: the Crimson Snapper (*L. erythropterus*, GCA_020091685.1^11^, 521 scaffolds) and the Mangrove Red Snapper (*L. argentimaculatus*, GCA_034769285, chromosome-level). This study presents the first chromosome-level, annotated genome of the Vermilion Snapper. This assembly will serve as a valuable resource for managing this commonly harvested species, for which basic biological knowledge is limited.

## Methods

### Sample acquisition

Muscle and gill arch tissue samples were obtained from one individual caught by hook and line at the Diaphus Bank site off Louisiana, United States (latitude 28.08595, longitude −90.6999, at a depth of 82.3 m; Supplementary Figure S1), while aboard the RV Southern Journey on 24 August 2019 (BioSample accession SAMN 40577064). Once onboard the ship, the tissues were stored in 95% ethanol or flash-frozen and then stored at −80 °C at the Laboratory of Analytical Biology, National Museum of Natural History, Smithsonian Institution. Genomic DNA was isolated from muscle using a Qiagen tissue genomic DNA extraction kit and quantified with the Quanti-IT dsDNA Broad Range Assay kit (Thermo Fisher Scientific) and the Qubit 4.0 Fluorometer. DNA integrity was assessed using 2.0% agarose gel electrophoresis.

### Short-read whole genome library construction and sequencing

A DNA aliquot was sent to Quick Biology Inc. for whole-genome library construction and short-read Illumina sequencing, hereafter referred to as WGS. The gDNA was sheared to approximately 300 bp using a Covaris M220 (Covaris, Woburn, MA). Library preparation was conducted using a KAPA HyperPrep kit (KAPA Biosystems, Wilmington, MA) with 10 ng of DNA as input. The final library quality and quantity were analyzed by Agilent Technologies 4200 station and a Qubit 3.0 Fluorometer (Thermo Fisher Scientific Inc., Waltham, MA). 150 bp paired-end libraries were sequenced on Illumina HiSeqX (Illumina Inc., San Diego, CA) for a target coverage of 100X.

### *Hi-C* Library construction and sequencing

A flash-frozen white muscle tissue sample was sent to Quick Biology Inc. for high-throughput chromosome conformation capture (Hi-C) library construction and short-read Illumina sequencing. Proximally ligated DNA was generated with the Arima High Coverage Hi-C Kit (Arima Genomics, Carlsbad, CA) using 130 mg of tissue as input. The library was prepared using the Arima library prep module (Arima Genomics, Carlsbad, CA) with an insert size of approximately 400 bp. The final library quality and quantity were analyzed by Agilent Technologies 4200 Tape Station (Agilent, Santa Clara, CA) and Qubit 3.0 Fluorometer (Thermo Fisher Scientific Inc., Waltham, MA). 150 bp paired-end libraries were sequenced on the Illumina NovaSeq. 6000 (Illumina Inc., San Diego, CA), aiming for a target coverage of 50X.

### *HiFi* Whole genome library construction and sequencing

A flash-frozen white muscle tissue sample was shipped to the Brigham Young University DNA Sequencing Center for PacBio HiFi library construction and long-read sequencing. Genomic DNA was isolated using the Qiagen Genomic-tips procedure and sheared with a Megaruptor. A HiFi SMRTbell library was prepared using a SMRTbell Library Kit according to the recommended protocols (PacBio, CA). Sequencing was performed on a Revio cell for 24 hours.

### *De novo* genome assembly

**Fig. 2:**
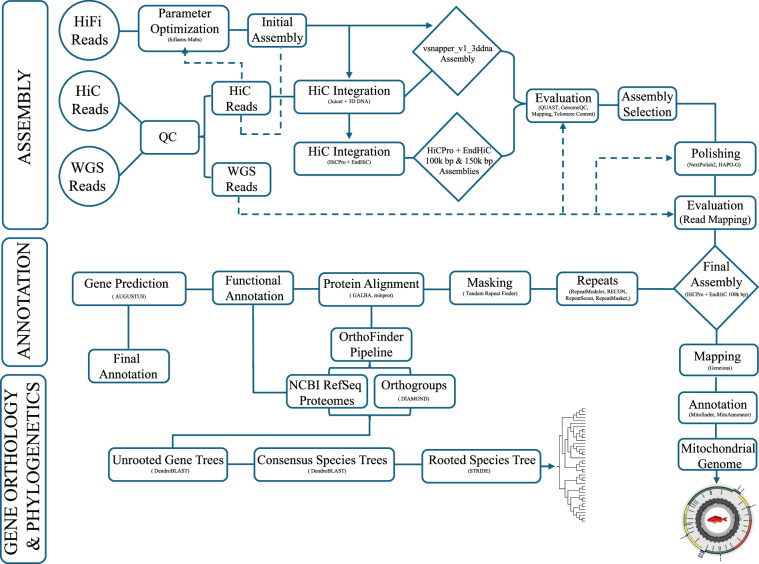
Workflow for genome assembly, annotation, gene orthology, and phylogenetics.

The assembly’s first draft was generated using hifiasm v.0.19.5^12,13^ using PacBio HiFi long reads in conjunction with Hi-C reads. The Mabs v2.19 package^14^ was used to assess the optimal parameter set for hifiasm by running several iterations with different parameters and measuring the performance of each intermediate assembly by using the Actinopterygii BUSCO^15^ database to estimate the number of single-copy orthologous genes present. The initial assembly (vsnapper_v1_hifiasm.fasta) included 24 chromosomal-level contigs. The analytical pipeline for generating the de novo assembly of Vermilion Snapper is illustrated in Fig. 2, and the pipeline is available on GitHub.

Two Hi-C data preprocessing and genome refinement pipelines were used to improve on the draft genome: (1) Juicer v.1.6^16^ followed by 3D-DNA release 201008^17^, and (2) HiC-Pro v.3.1.0^18^ followed by EndHiC v.1.0^19^. First, the draft genome was indexed with bwa^20^. Four restriction sites were then used to digest the assembly using generate_site_positions.py from Juicer. The output file was processed with Juicer using the default parameters, resulting in the Hi-C reads required for the 3D-DNA pipeline. The 3D-DNA pipeline was run with default parameters, except for the “refinement” rounds step (–rounds parameter), which was set to three rounds instead of two. Following the fragmentation of the initial assembly to correct errors in the Hi-C data, the 24 chromosomes were manually assembled and curated using Juicebox. Subsequently, the drafted chromosomes were reassembled, and the remaining contigs were corrected as part of the post-curation step of the 3D-DNA pipeline. This process utilized a combination of Juicer and 3D-DNA pipelines, resulting in a single assembly (vsnapper_v1_3ddna.fasta).

During the execution of the second preprocessing pipeline, the draft genome generated with hifiasm was used with the digest_genome.py script from HiC-Pro software to digest the genome with four restriction enzymes. Then, Hi-C link matrices were created using HiC-Pro restriction-digested genome and the quality-controlled Hi-C reads. Default parameters were used except bin size was set to (BIN_SIZE = 25000 50000 100000 150000 200000 500000 1000000) and ligation site was set to (LIGATION_SITE = GATCGATC, GANTGATC, GANTANTC, GATCANTC) according to Arima Hi-C kit manufacturer instructions. The output matrices were then input into EndHiC with 100000 and 150000 bin sizes, which resulted in two assemblies (vsnapper_v1_endhic_100k.fasta and vsnapper_v1_endhic_150k.fasta).

The four assembly versions (hifiasm, 3D-DNA, EndHiC –100 K, and EndHiC –150 K) were evaluated using QUAST v.5.2.0^21^. Telomere content was also assessed based on patterns for fish from the Telomerase Database and from the chromosome-level genome assembly of *Nibea coibor*^22^ using facilities from the Bioconductor (release v3.18) package Biostrings (v.2.70.3) while ggplot2 (v.3.4.2) in R v4.3.1. Specifically, the telomere patterns TTAGGG and CCCTAA were searched across the assembled chromosomes. Their appearance frequency was evaluated using histograms and density plots (Supplementary Figure S2), revealing higher occurrence at the edges of assembled chromosomes (telomere evaluation scripts available on GitHub).

Additionally, GenomeQC^23^ was used to evaluate the assemblies, and BUSCO for gene content. Based on the results (see below), the best assembly (vsnapper_v1_endhic_100k.fasta) was then subjected to polishing for small structural error correction using long reads (NextPolish2 v.0.2.0^24^) and short WGS reads (HAPO-G v.1.3.7^25^). Additionally, the Juicer + 3D-DNA assembly was also polished for further inspection of the first chromosome.

### Genome annotation

Repetitive elements in the vsnapper_v1_endhic_100k.fasta genome assembly were identified *de novo* using RepeatModeler v.2.0.5, which includes the programs RECON v.1.05 and RepeatScout v.1.06^26^. Repetitive elements were classified using RepeatClassifier v.2.0.5^27^ and soft-masked using RepeatMasker v.4.1.5^27^, resulting in 39.16% of the assembly being masked. Additional masking was performed using the Tandem Repeat Finder v4.09.1^28,29^.

The masked genome assembly was annotated using the GALBA v.1.8.9 pipeline^30^. GALBA aligns reference proteins to the genome assembly with minprot^31^ and uses the alignments to predict genes with AUGUSTUS^32,33^. Reference proteins were obtained from the NCBI RefSeq database^34^ proteomes of 38 fish species in the Eupercaria clade (Table S6).

### Gene orthology and phylogenetic analyses

We assessed the quality of the predicted protein set for our assembly by comparing BUSCO v5 scores with those from the RefSeq proteomes using the Actinopterygii ortholog set as described above. Orthologous genes and phylogenetic inferences among Eupercaria fish species were performed with the OrthoFinder pipeline v.2.5.5^35,36^. In short, the amino-acid sequences were grouped by similarity into Orthogroups with DIAMOND v2.1.9.163^37^^,^ and gene trees were inferred for each orthogroup using the alignment-free method implemented in DendroBLAST 2.15.0+^38^. Gene trees were used to infer a consensus species tree using STAG v.1.0.0^39^. STAG branch support values represent the proportion of individual species trees that contain a given speciation event. The consensus species tree was rooted with STRIDE v.1.0.0^40^.

### Assembly and annotation of the mitochondrial genome

We assembled the mitochondrial genome of *Rhomboplites aurorubens* in Geneious 2023.2.1 (https://www.geneious.com). We used a complete mitogenome previously sequenced from the same species (NCBI accession number: NC_082561.1^41^) as a reference sequence for the assembly of long reads, and Map to Reference, using the same parameters for a single set of up to 10 iterations. The MitoAnnotator tool from the MitoFish Mitochondrial Genome Database of Fish^42^ was used to annotate genes, ribosomal RNAs (rRNAs), and transfer RNAs (tRNAs).

## Data Records

This whole genome project has been deposited at DDBJ/ENA/GenBank under the accession JBFPGM010000000. The version described in this paper is version JBFPGM000000000^43^. Genome raw reads have been deposited in the National Center for Biotechnology Information (NCBI) Sequence Read Archive (SRA)^44–46^ under BioProject PRJNA1090878, and the genome assembly is associated with the same BioProject under the “container” BioSample SAMN40577064. GenBank accession number for the mitogenome nucleotide sequence is PQ210872 (BankIt2851790 ptg000083l_polished). Scripts, genome and mitogenome assembly and annotation files, figures, tables, and supplementary material were deposited in the Smithsonian Institution research repository, figshare database^47^.

## Technical Validation

### Genome sequencing, assembly, and evaluation

**Fig. 3:**
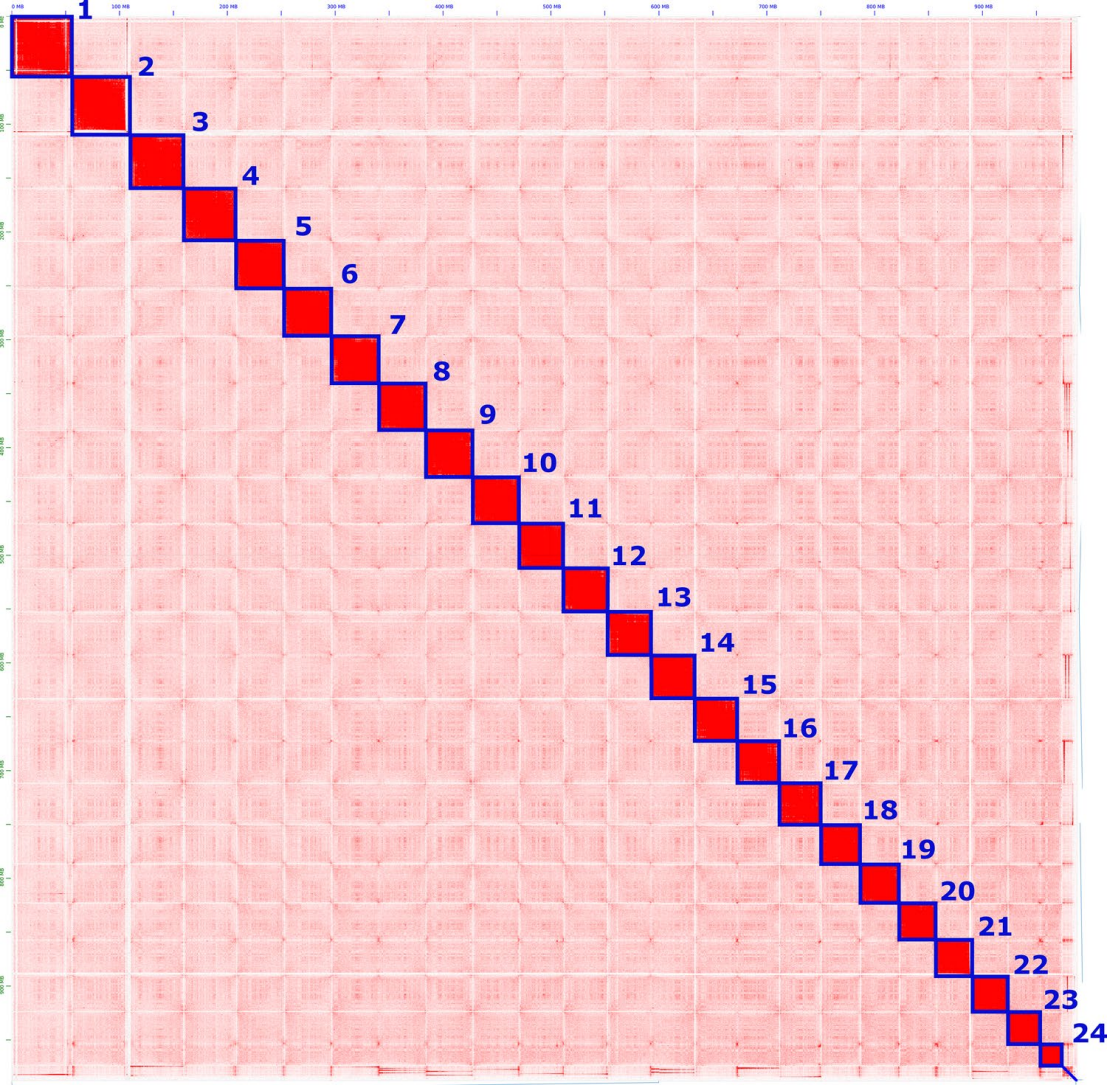
HiC contact and assembly map of *Rhomboplites aurorubens* after curation. The blue squares represent chromosomes assembled by hand using the Juicebox view.

The Vermilion Snapper data included 6.7 Mbp PacBio long reads (Table 1), 224.2 Mbp Hi-C reads, and 316.8 Mbp WGS short reads (Table 2, Supplementary Table S2). The results from QUAST suggested that, despite 3D-DNA being slightly longer, it contained a lot of Ns. In contrast, assemblies generated with the HiC-Pro + EndHiC pipeline (100k bp and 150k bp) showed higher assembly quality statistics (Supplementary Table S3, Figure S3). The spatial organization of the chromosomes was visualized in Juicebox, confirming that the Hi-C integration with three rounds was adequate (Supplementary Figure S4). Results from the telomere content analysis indicated that the first contig (“chromosome 1”) is generally better resolved using the Juicer + 3D-DNA pipeline and manual intervention (Fig. 3). However, the best overall results were from the HiC-Pro + EndHiC pipeline at the 100k bp bin scale. Evaluation of the three main assemblies (snapper_v1_3ddna, HiC-Pro + EndHiC pipeline 100k bp, HiC-Pro + EndHiC pipeline 150k bp) using Genome QC showed that all assemblies contained >97.5% completeness and were single-copy BUSCO genes (Supplementary Figure S5). Based on the methods used to evaluate the quality of the assemblies, the assembly from HiC-Pro + EndHiC pipeline at 100k bp bin size was the highest quality assembly. This assembly was used for further polishing for minor structural error corrections using long reads (NextPolish2) and short WGS reads (HAPO-G). The four assembly versions were also evaluated with short read mapping (Fig. 2). The number of paired WGS reads and bases for each unpolished and polished genome assembly are detailed in Supplementary Tables S4, S5. The final, polished assembly version of the HiC-Pro + EndHiC pipeline, at a 100k bp resolution, was used for annotation and downstream analyses. The assembly initially comprised 135 contigs, from which 20 were removed because they contained mitogens before being uploaded to GenBank. The final assembly was ordered onto 24 chromosomes with a total length of 987.5 Mbp and a contig N50 of 41.3 Mbp (Table 3).

**Table 1:** HiFi sequencing data statistics.

PacBio HiFi	Total	Failed assigned	Failed unassigned	HiFi assigned	HiFi unassigned
Reads	6,995,717	250,577	66,192	6,440,358	238,590
Bases	6,995,717	3,488,518,100	911,239,713	87,907,918,772	3,277,966,427

**Table 2:** Hi-C and Whole Genome Sequencing data statistics.

Reads	Read pairs	Bases per pair	%GC
Illumina HiC	235,149,204	35,107,471,426	40
Illumina WGS	332,230,301	46,059,753,867	41

**Table 3:** Summary of genome assembly statistics, gene annotation statistics, and BUSCO completeness.

Basic Statistics	Vermilion Snapper
Chromosome assembly size	987486130 bp
Non-ATGC characters	8.10E-08
GC content	40.50%
Mean base-level coverage	59.02x
Chromosome-scale N50	4.1 Mb
Contig N50	40.5
BUSCO genome completeness	97.50%
Number of protein-coding genes	34496
BUSCO gene annotation completeness	97.80%
Complete and single copy	83%
Complete and duplicated	14.80%
Fragmented	0.60%
Missing	1.60%

### Genome annotation, gene families, and phylogeny

**Fig. 4:**
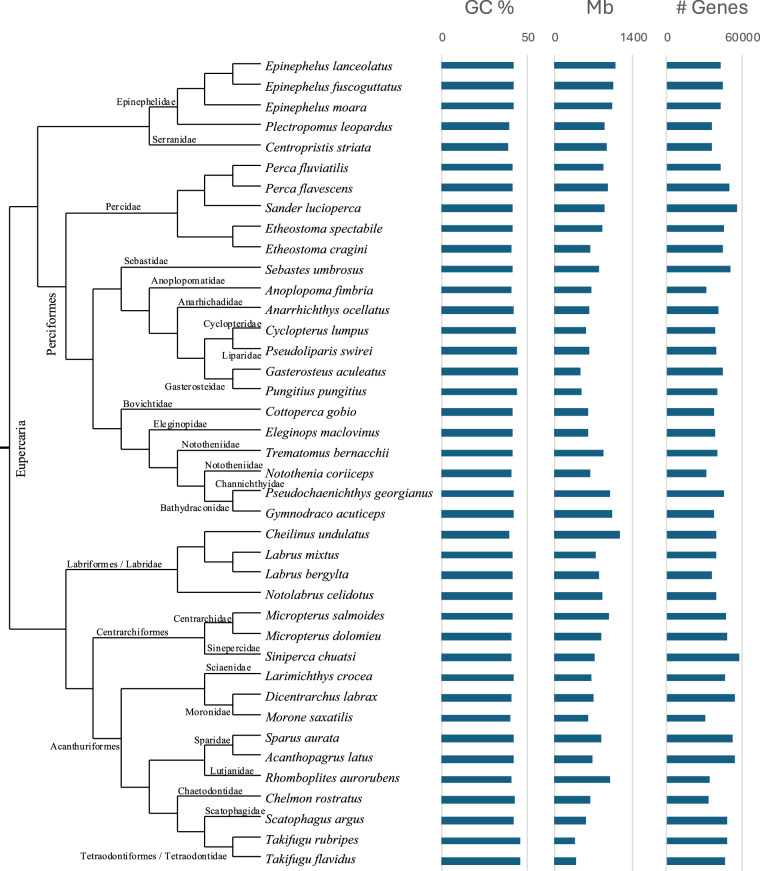
Species Tree inference from All Genes (STAG) analysis of *Rhomboplites aurorubens*, the Vermilion Snapper genome. The GC content (%GC), genome size (Mb), and number of genes are presented as barplots.

The quality of the genome assembly of Vermilion Snapper is comparable to other Eupercaria assemblies (Supplementary Table S6). The Vermilion Snapper has a contiguity (N50) of 41.3 Mbp, which is greater than most of the other fish genomes in our comparison (0.2–41.1 Mbp), except for representatives of the families Epinephelidae, Channichthyidae, and Labridae (42.8–51.5 Mbp). The completeness of the Vermilion Snapper assembly (97.8% complete metazoan orthologs, including single-copy and duplicated) is one of the most complete fish genomes sequenced to date (78.8–99.9%). The GC-content of the species ranges between 38.5% and 45.5%, with an intermediate value of 40.5% for Vermilion Snapper. The assembly size is also among the largest (987.5 Mbp) but contains the fewest predicted genes, among those assessed (Fig. 4, Table S6).

The Eupercaria is one of the most diverse groups of fishes, comprising over 7,000 species, yet resolving its phylogenetic relationships has remained challenging. Our Eupercaria species tree phylogeny, including single-copy orthologs present in all species, supports two major clades: Perciformes and a lineage comprising Centrarchiformes, Labriformes, Acanthuriformes, and Tetraodontiformes (Fig. 4, classification following^48^). Although taxon sampling is limited, and testing hypotheses regarding Eupercaria is beyond the scope of this study, our results are mostly congruent with recently published phylogenies^48–51^. However, within the Perciformes clade, most phylogenetic hypotheses differ on how families (Labridae, Scianidae, Moronidae, Sparidae, and Lutjanidae) are related^49–51^. In the particular case of Lutjanidae, the family including the Vermilion Snapper, has been placed within either order Acanthuriformes^48,49,51^ or Lutjaniformes^50^; our results support the latter. Discrepancies among major published phylogenetic classifications likely stem from taxonomic and molecular sampling differences. As the growing body of genomic data informs the fish tree of life, we can expect more robust and consistent classifications.

### Mitochondrial genome assembly and annotation

**Fig. 5:**
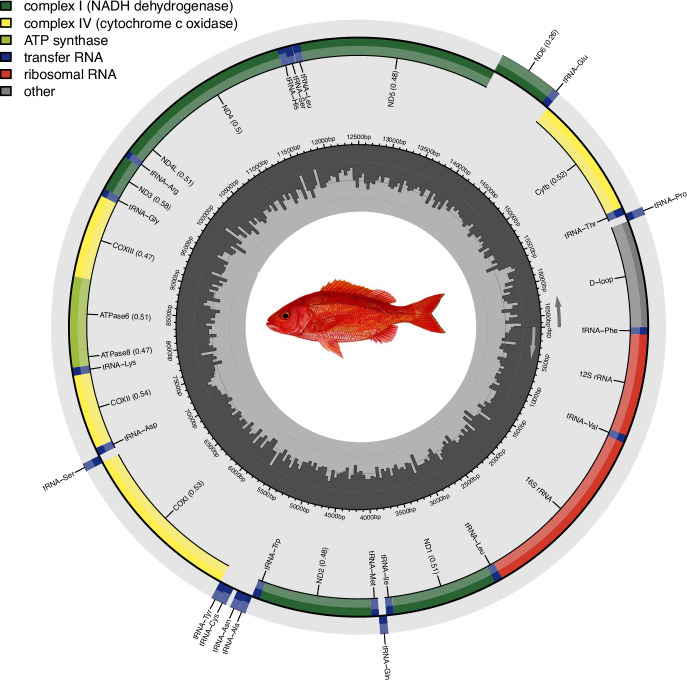
Mitochondrial genome assembly and annotation of *Rhomboplites aurorubens*. Dark grey bars (inner track): GC skew. Bars extending outward from the dashed baseline are positive skew (G > C), and bars extending inward are negative skew (G < C).

The mitochondrial genome resulted in a 16,670 bp circular mitogenome. The mitogenome is similar in length to the two previously published *R. aurorubens* mitochondrial genomes (NCBI accession numbers: NC_082561.1 and PP033003.1), with 99.61% and 99.5% sequence identity, respectively (16,506 and 16,502 bp of 16,670 bp), as calculated by BLASTn v2.10.0^52^. In total, 13 genes, 2 RNAs, and 22 tRNAs were annotated using the MitoAnnotator tool from the MitoFish Mitochondrial Genome Database of Fish (Fig. 5).

In this study, we generated a complete chromosome-scale genome assembly of a Vermilion Snapper (*R. aurorubens)*. Although the Vermilion Snapper is a significant commercial and recreational species, it has remained understudied and is often mislabeled as Red Snapper, which undermines conservation efforts. In addition to providing a high-quality reference genome to further our understanding of its genomic architecture, this resource will serve as a reference for studies of population genomics, local adaptation, and responses to environmental variation. It will further improve our understanding of the biology of this species as well as that of other key species in the area.

### Ethics approval

This project was approved by the National Museum of Natural History’s (NMNH) Institutional Animal Care and Use Committee (IACUC; SI-21020), and all parts of this study have been conducted in accordance with the approved protocols and relevant guidelines and regulations.

## Supplementary information

Table S1

Table S2

Table S3

Table S4

Table S5

Table S6

Figure S1

Figure S2

Figure S3

Figure S4

Figure S5

## Data Availability

Software for data analyses were described in the Methods section. The genome assembly code is available at https://github.com/moulos-lab/vermilion-snapper-assembly. The genome annotation, the mitogenome assembly and annotation, and other supplementary material are accessible at https://smithsonian.figshare.com/projects/The_chromosome-level_genome_of_the_Vermilion_Snapper_Rhomboplites_aurorubens_/210715.
