# Supplementary figures and images for: Chromosome-level genome assembly of the Vermilion Snapper (Rhomboplites aurorubens)
Source: Sci Data. 2025 Jul 23;12:1281. doi: 10.1038/s41597-025-05573-w (PMC12287329; doi:10.1038/s41597-025-05573-w)

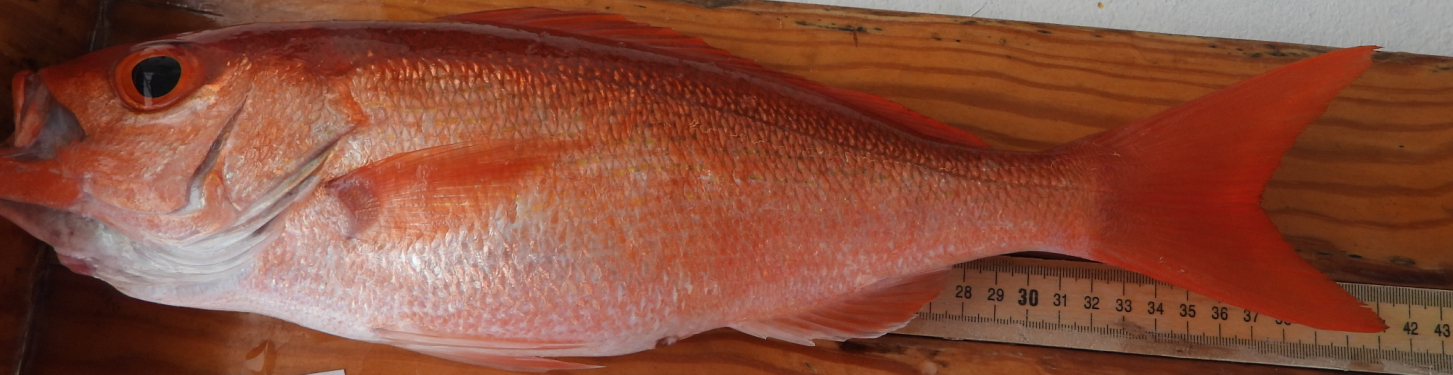

PE-2019-429

Supplement: Supplementary file 7 — Figure S1 [file 41597_2025_5573_MOESM7_ESM.pdf]

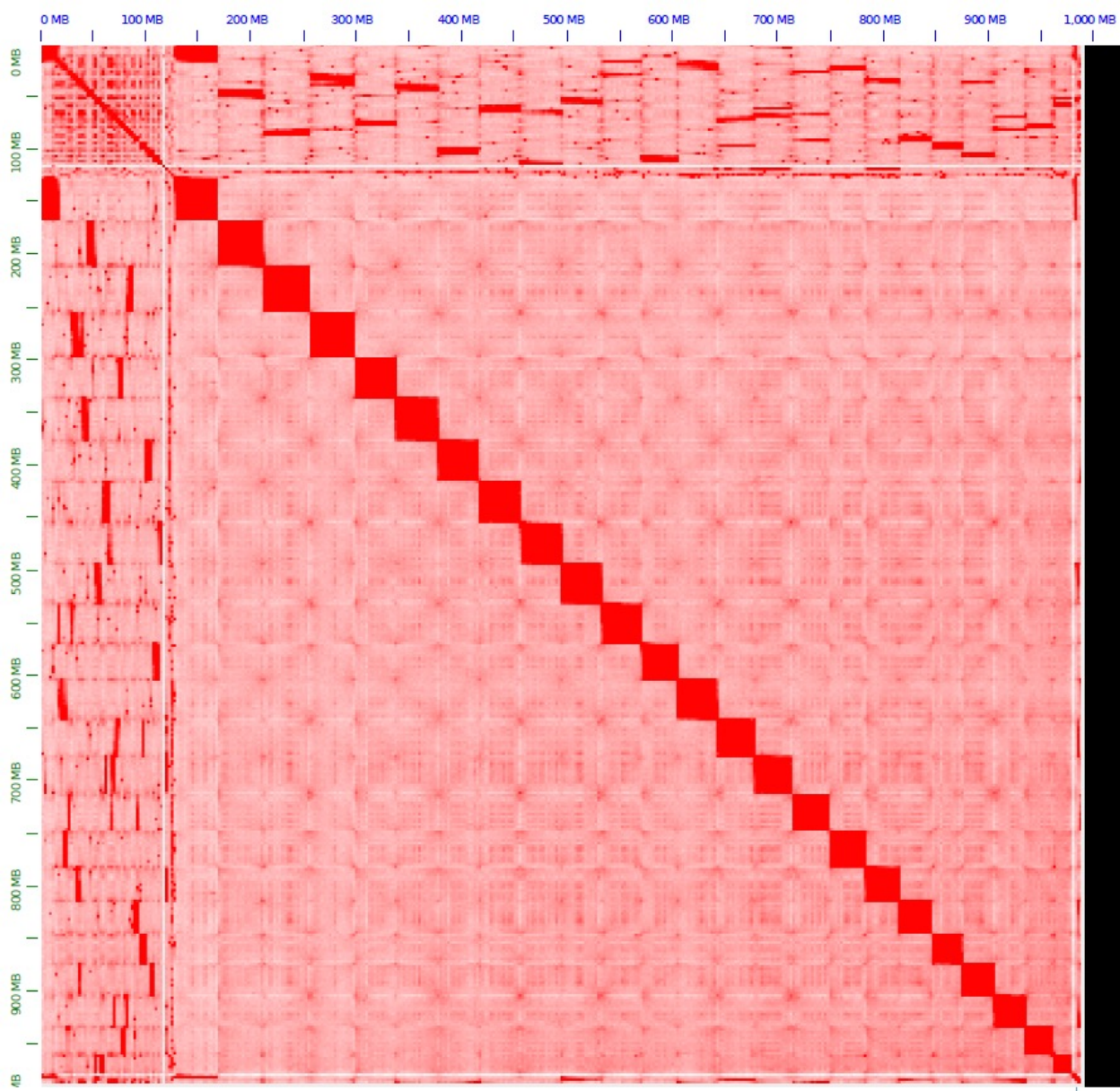

Supplement: Supplementary file 10 — Figure S4 [file 41597_2025_5573_MOESM10_ESM.pdf]

Busco Plot

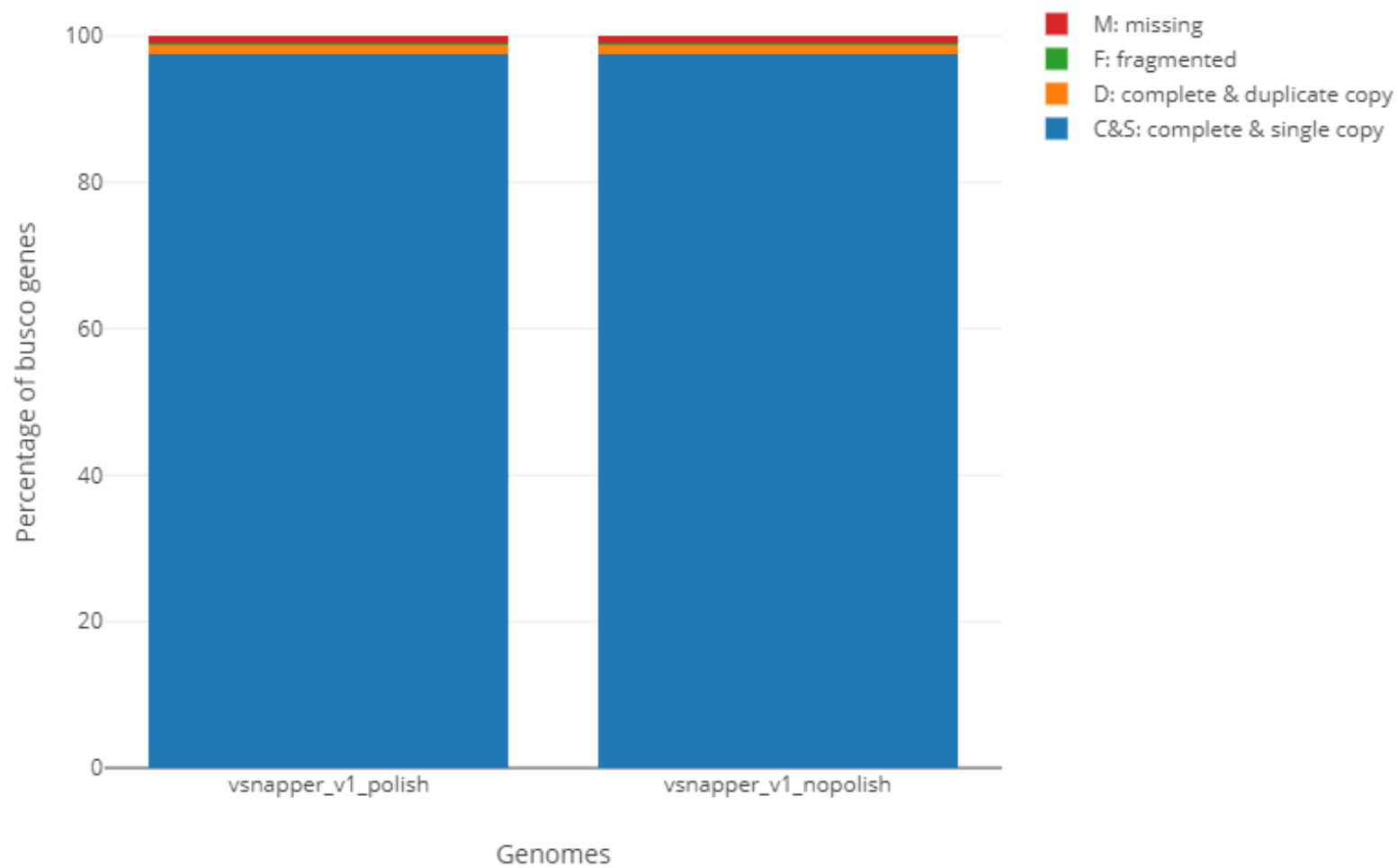

Supplement: Supplementary file 11 — Figure S5 [file 41597_2025_5573_MOESM11_ESM.pdf]
