# Supplementary material for: Chromosome-level genome assembly of the Vermilion Snapper (Rhomboplites aurorubens)
Source: Sci Data. 2025 Jul 23;12:1281. doi: 10.1038/s41597-025-05573-w (PMC12287329; doi:10.1038/s41597-025-05573-w)

Telomere sequence content

Pattern TTAGGG

Chr_01

Histogram of pattern occurrence frequency

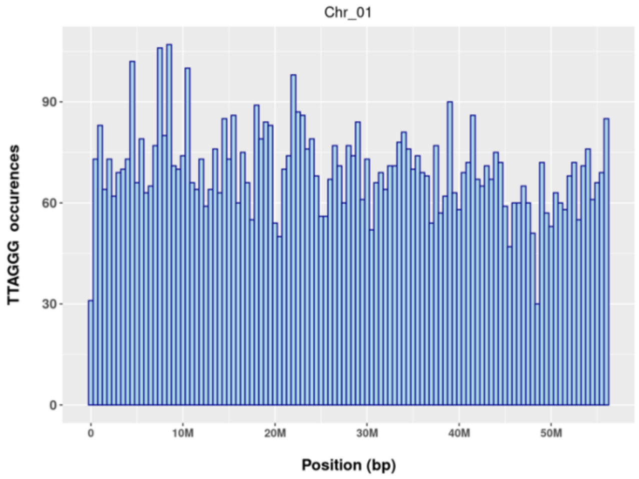

Density of pattern occurrence frequency

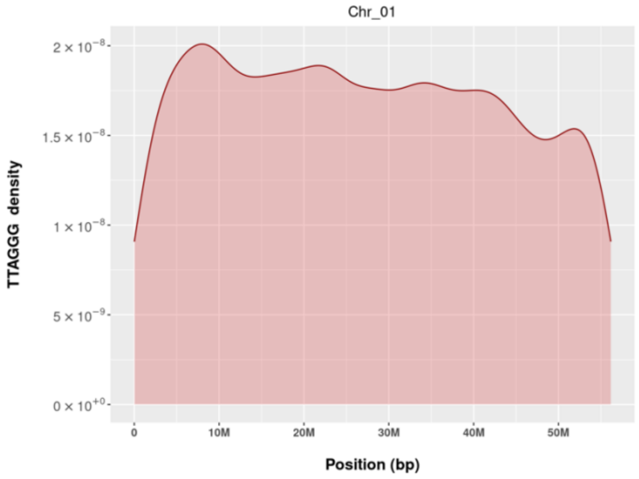

Chr_02

Histogram of pattern occurrence frequency

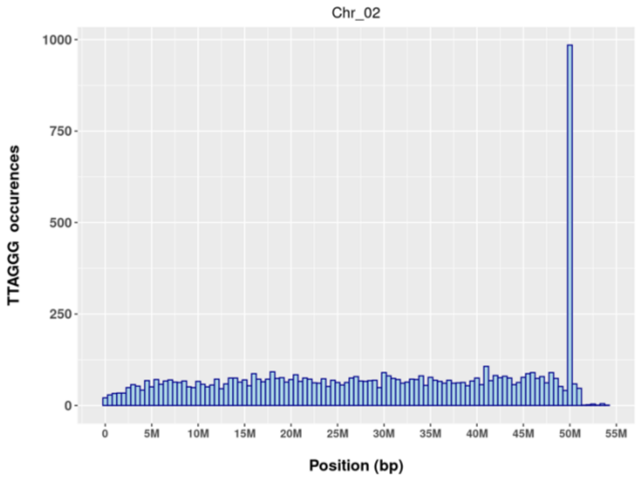

Density of pattern occurrence frequency

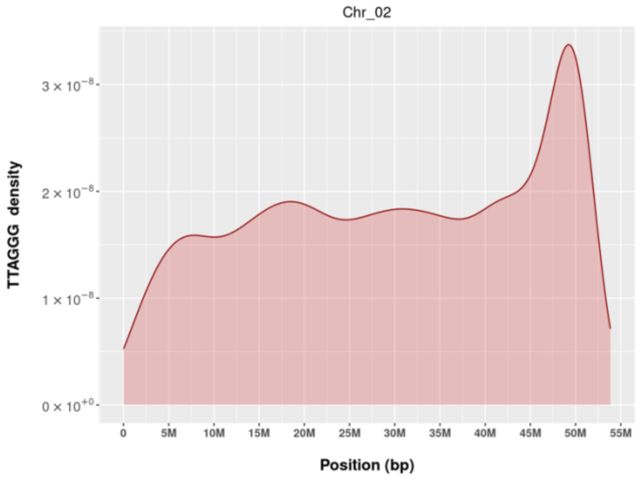

Chr_03
Histogram of pattern occurrence frequency

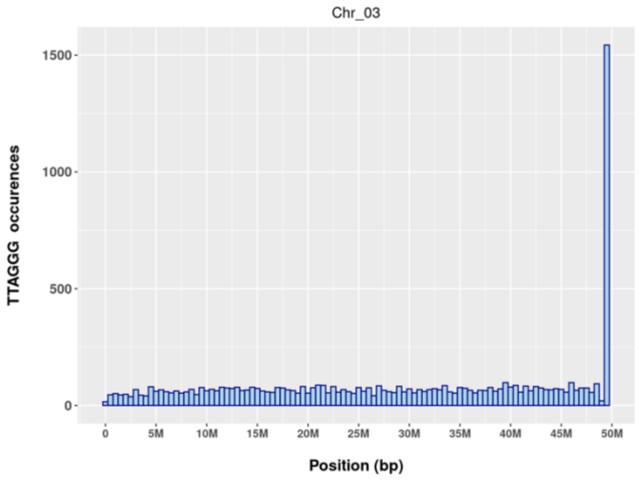

Density of pattern occurrence frequency

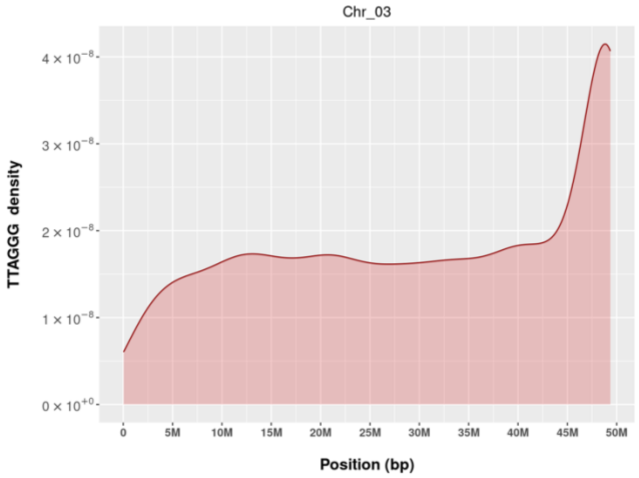

Chr_04

Histogram of pattern occurrence frequency

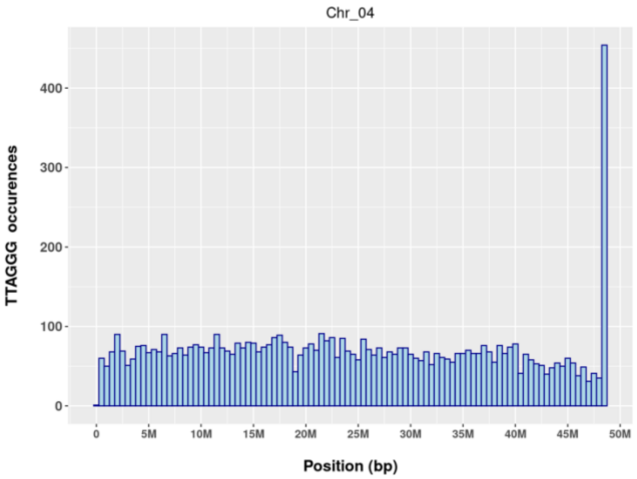

Density of pattern occurrence frequency

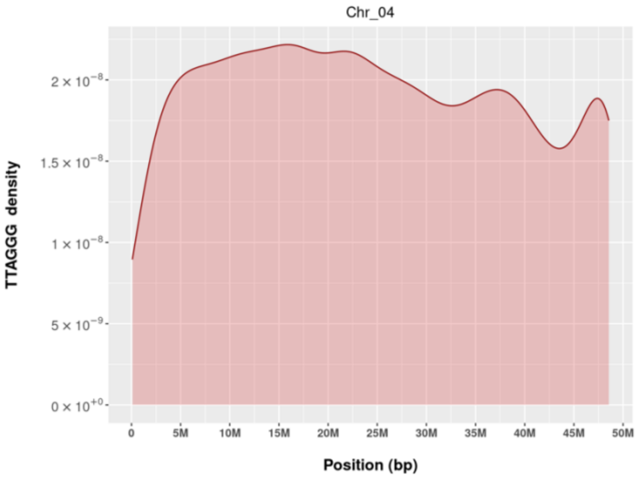

Chr_05

Histogram of pattern occurrence frequency

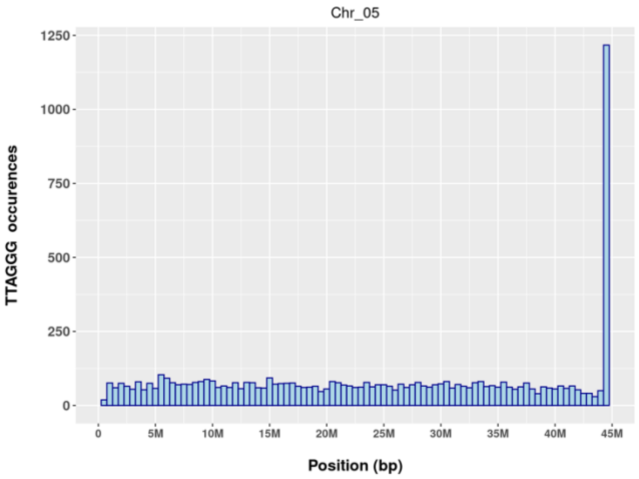

Density of pattern occurrence frequency

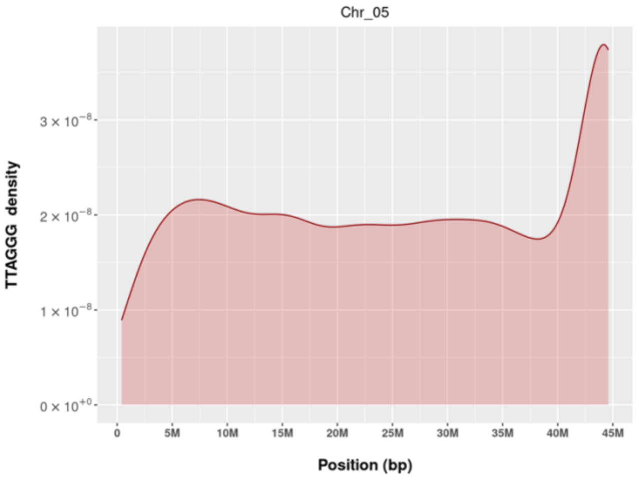

Chr_06

Histogram of pattern occurrence frequency

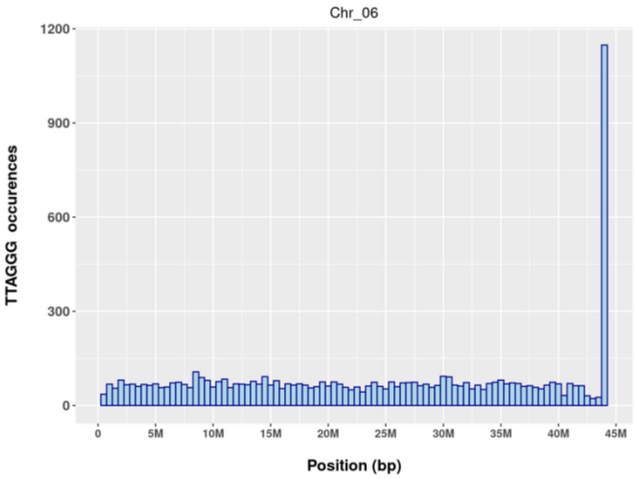

Density of pattern occurrence frequency

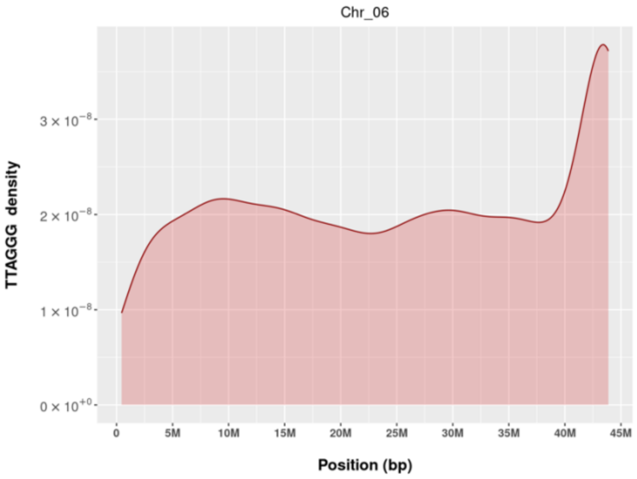

Chr_07

Histogram of pattern occurrence frequency

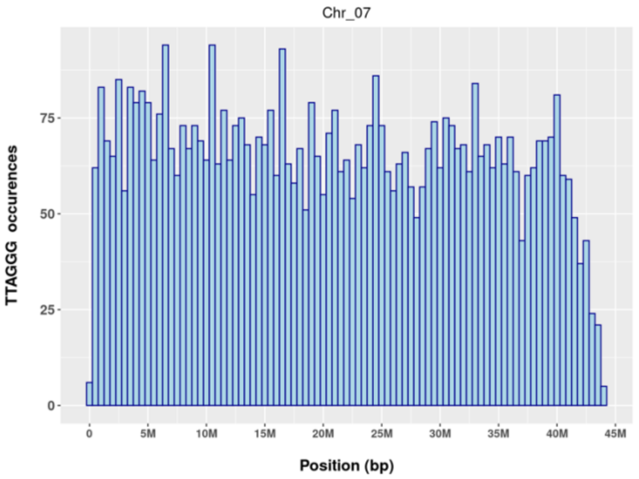

Density of pattern occurrence frequency

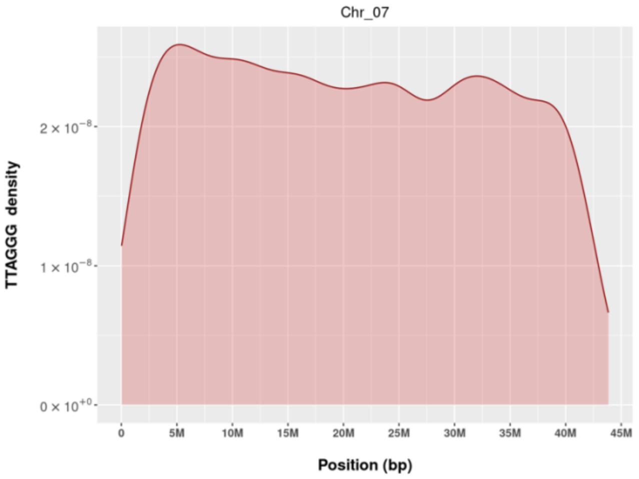

Chr_08

Histogram of pattern occurrence frequency

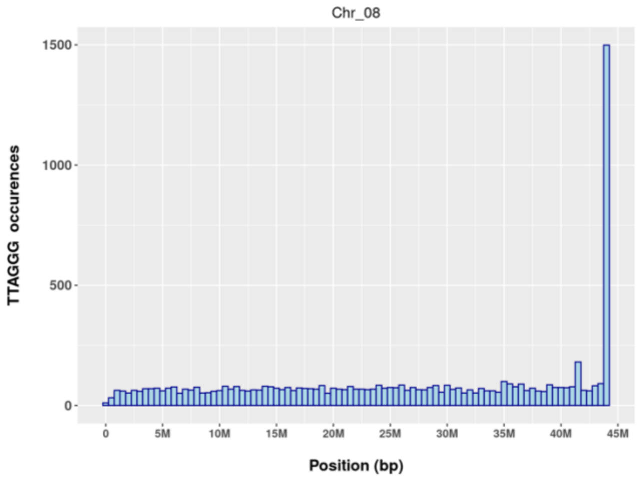

Density of pattern occurrence frequency

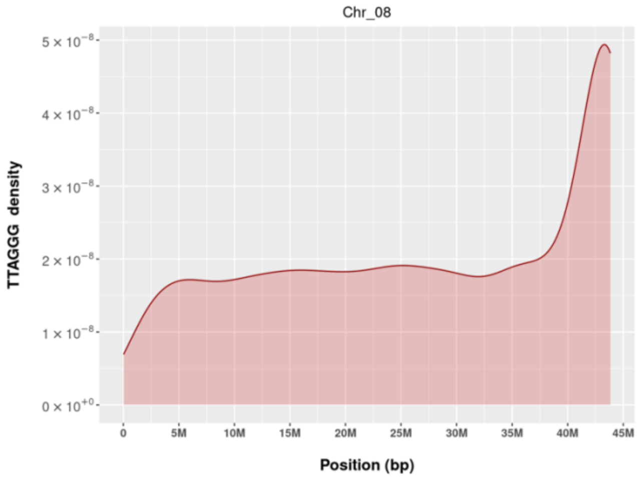

Chr_09

Histogram of pattern occurrence frequency

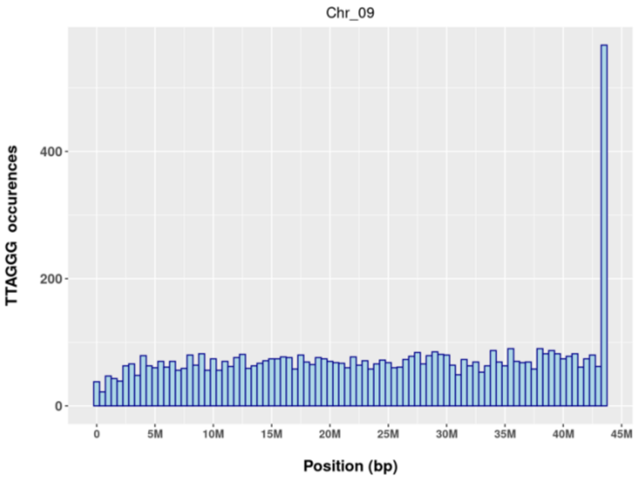

Density of pattern occurrence frequency

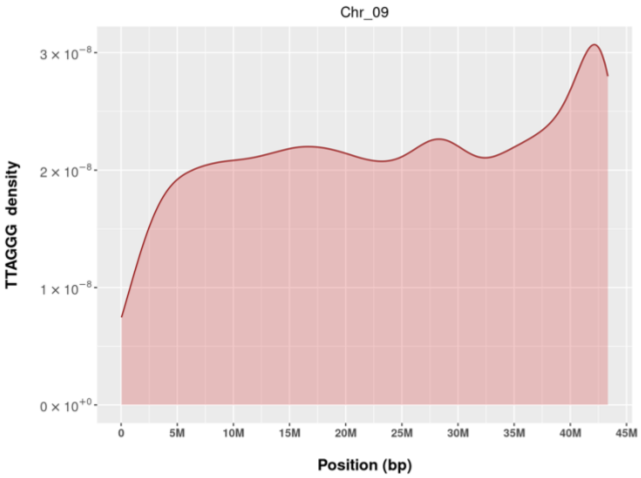

Chr_10

Histogram of pattern occurrence frequency

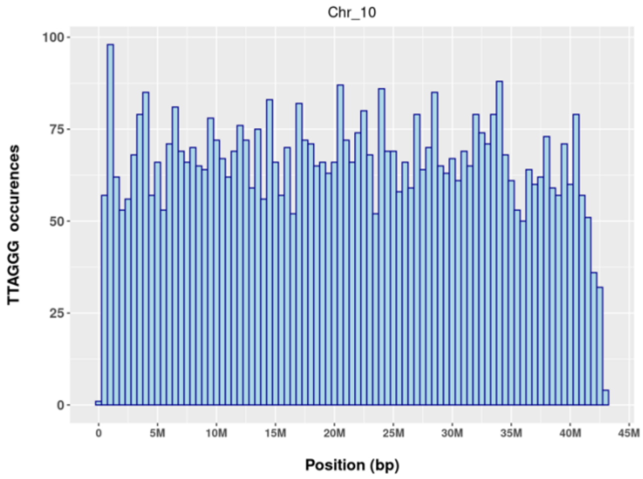

Density of pattern occurrence frequency

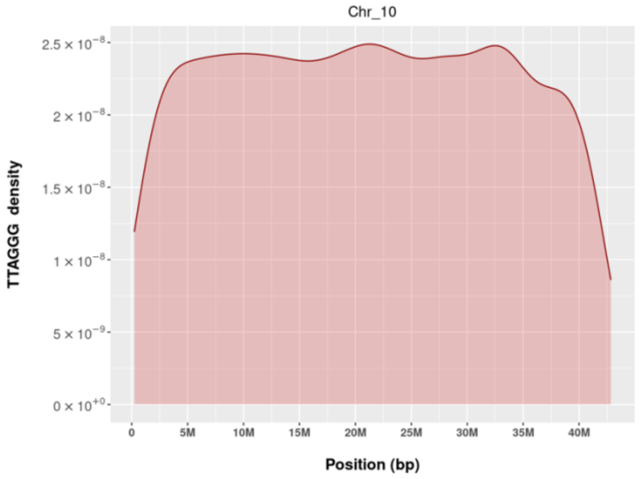

Chr_11

Histogram of pattern occurrence frequency

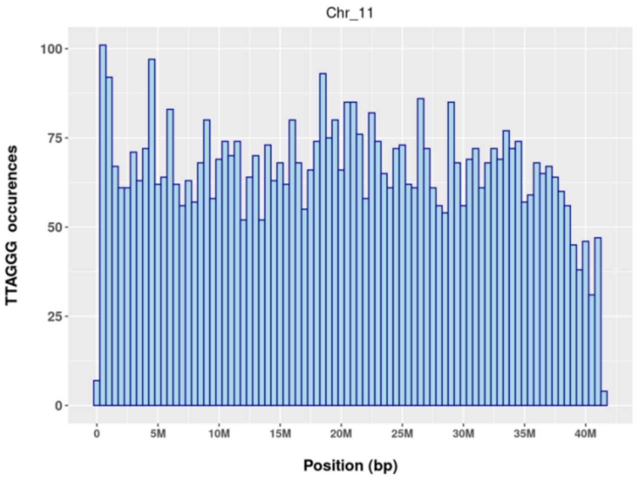

Density of pattern occurrence frequency

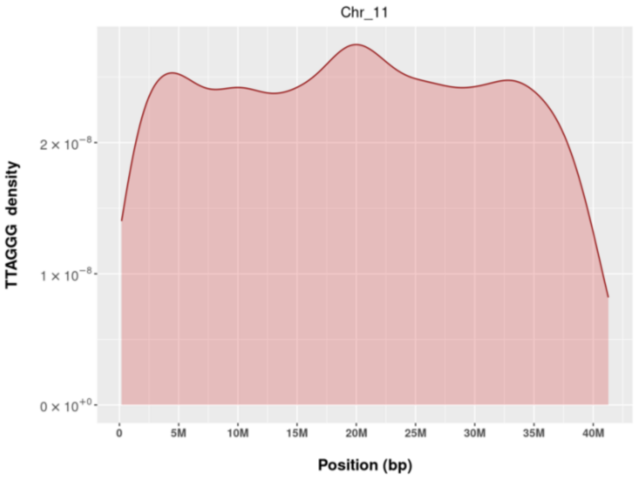

Chr_12

Histogram of pattern occurrence frequency

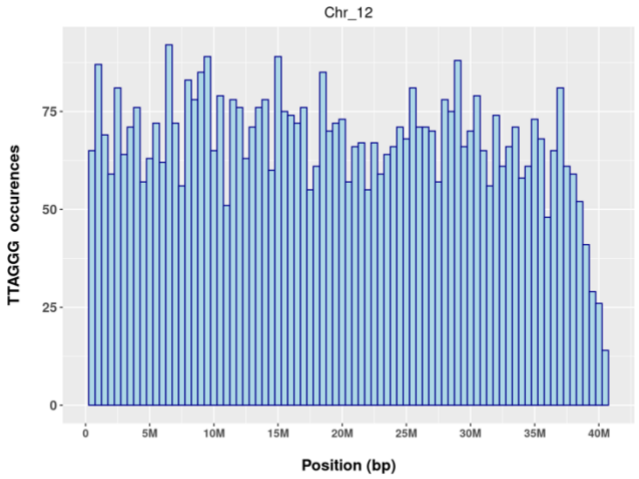

Density of pattern occurrence frequency

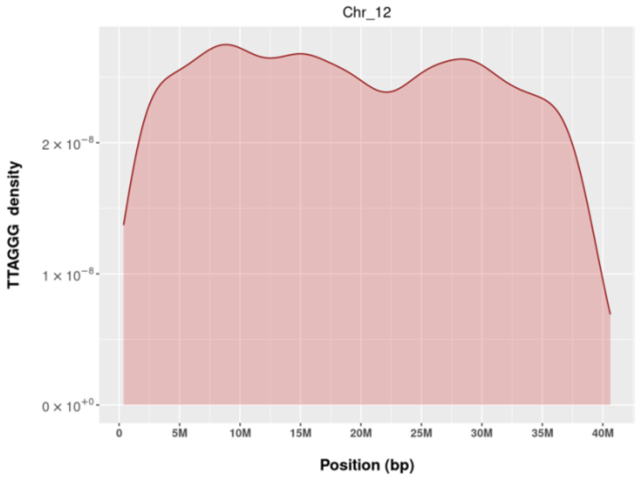

Chr_13

Histogram of pattern occurrence frequency

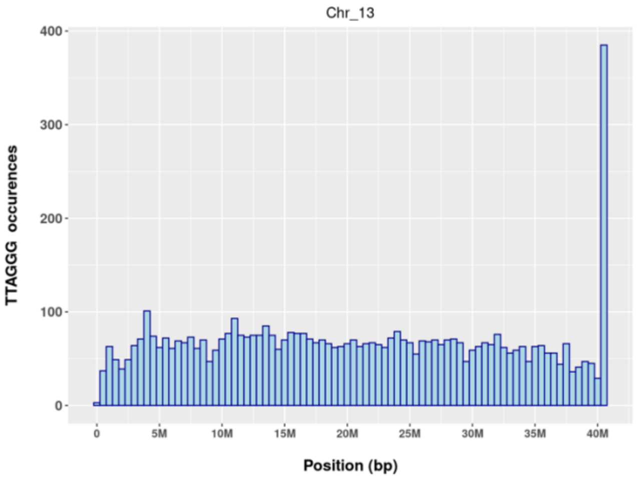

Density of pattern occurrence frequency

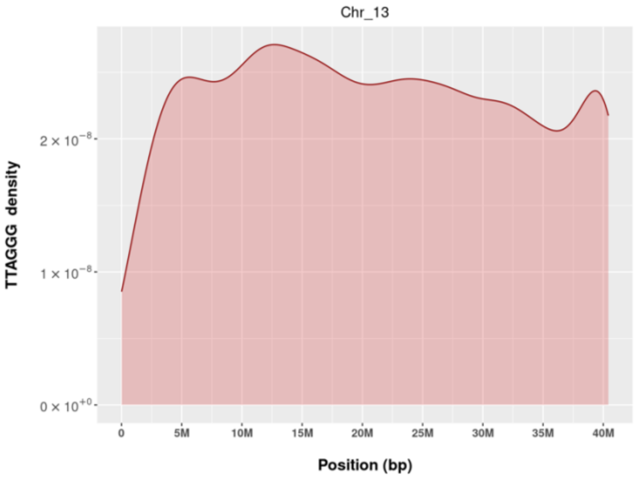

Chr_14

Histogram of pattern occurrence frequency

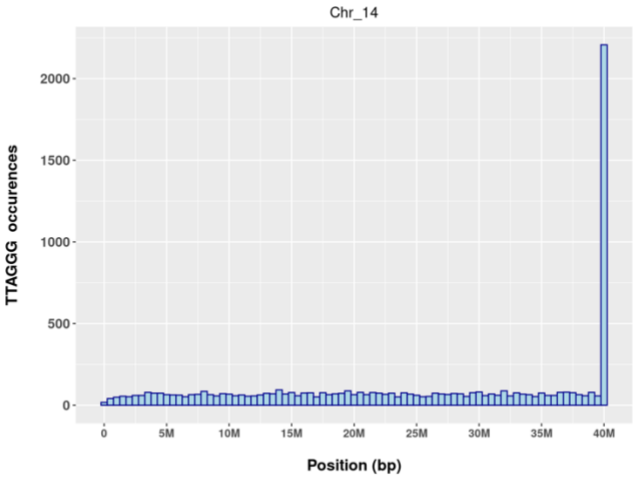

Density of pattern occurrence frequency

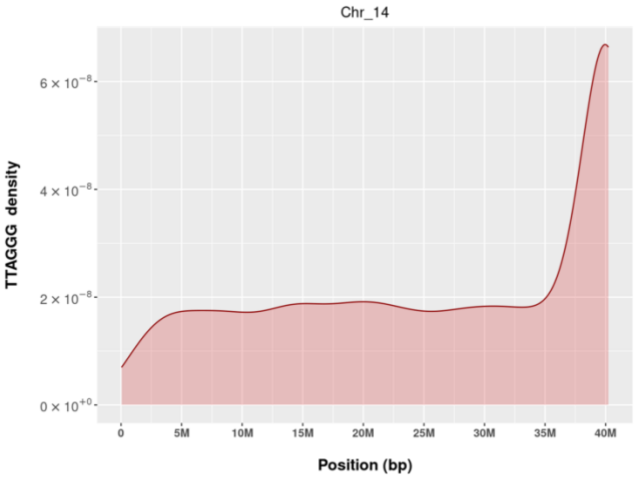

Chr_15

Histogram of pattern occurrence frequency

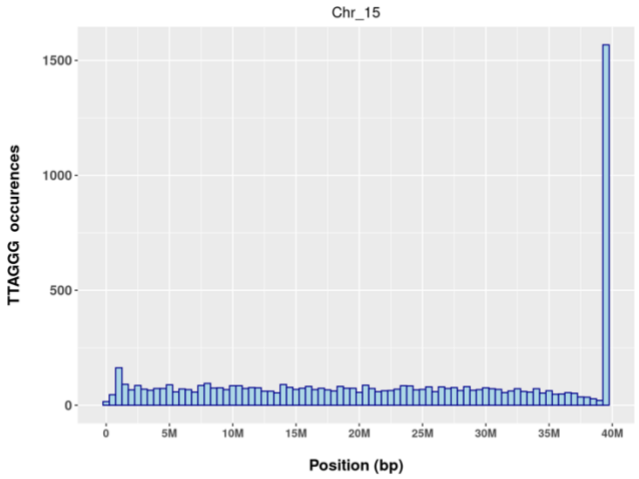

Density of pattern occurrence frequency

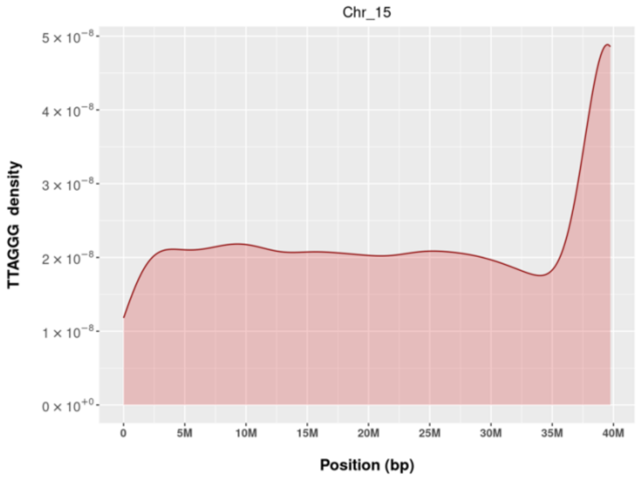

Chr_16

Histogram of pattern occurrence frequency

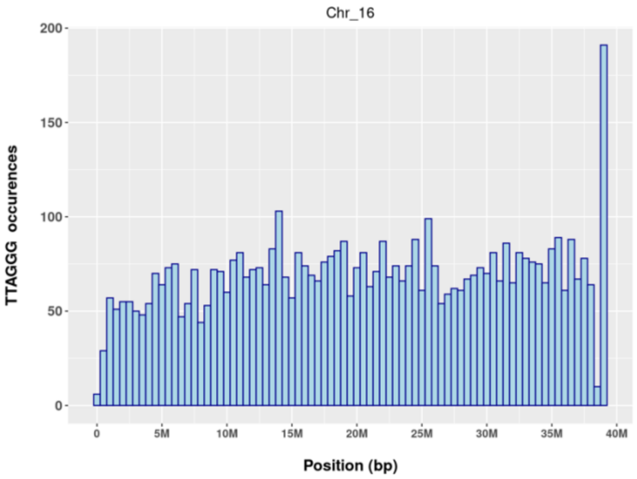

Density of pattern occurrence frequency

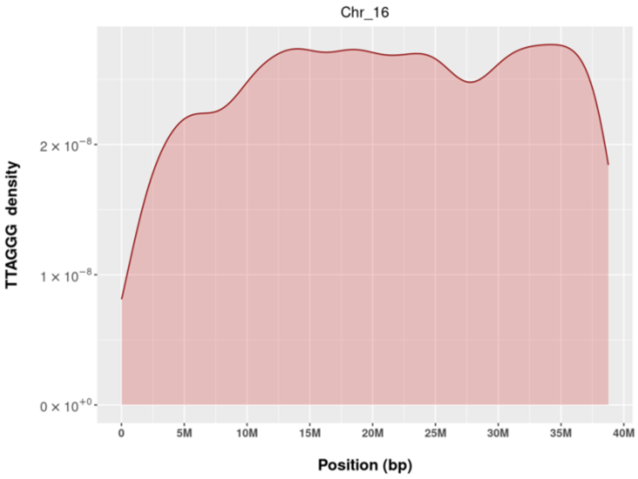

Chr_17

Histogram of pattern occurrence frequency

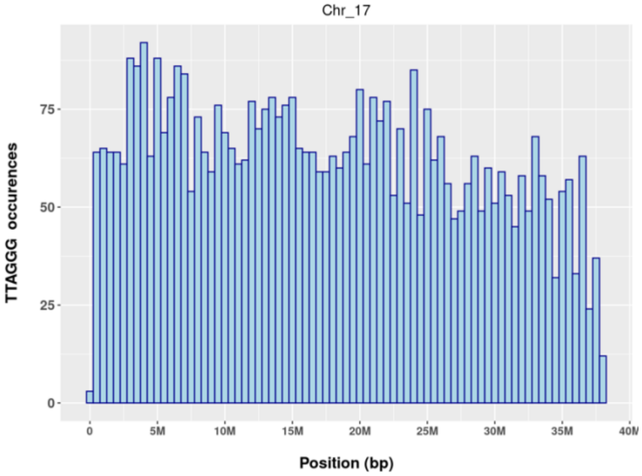

Density of pattern occurrence frequency

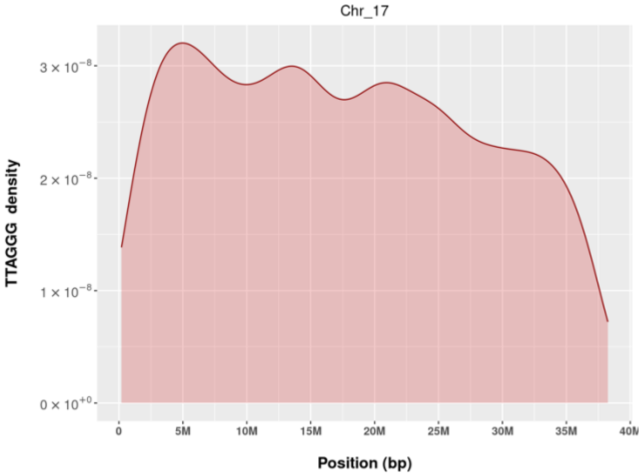

Chr_18

Histogram of pattern occurrence frequency

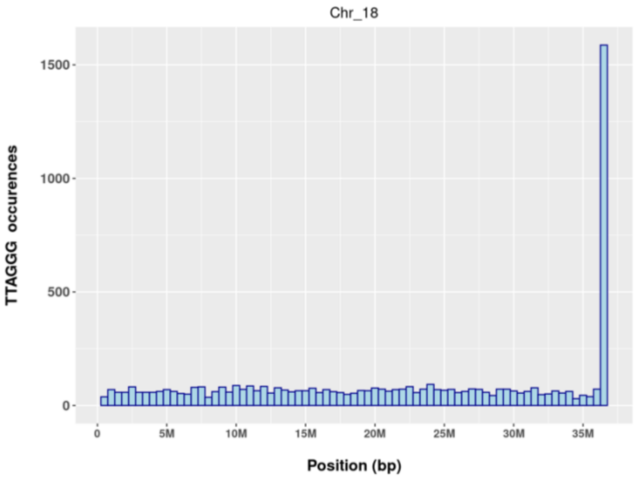

Density of pattern occurrence frequency

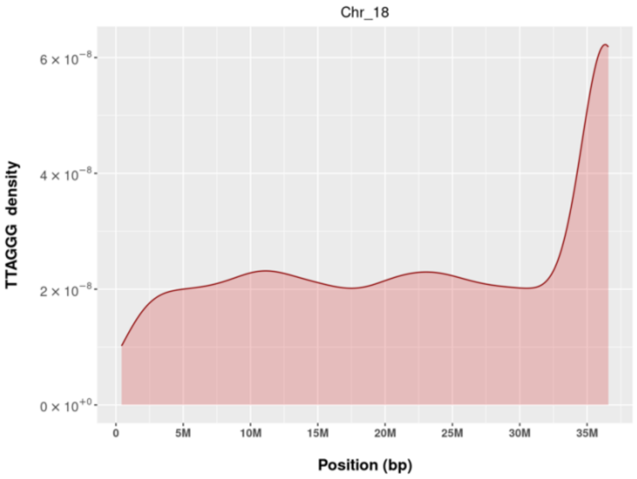

Chr_19

Histogram of pattern occurrence frequency

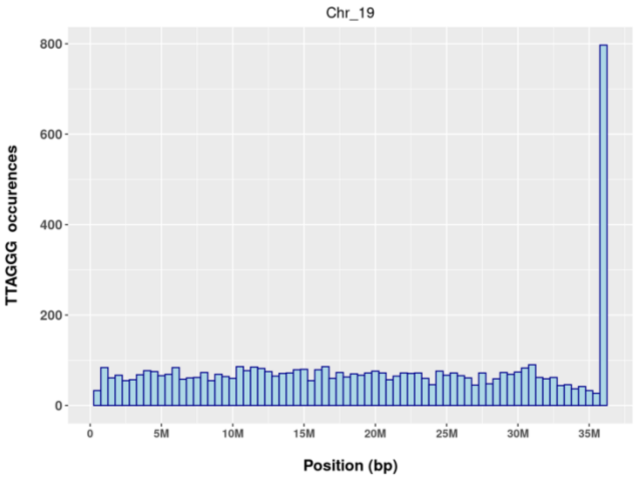

Density of pattern occurrence frequency

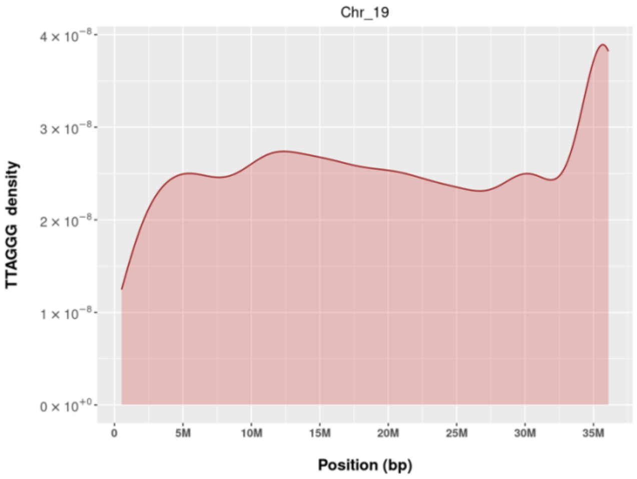

Chr_20

Histogram of pattern occurrence frequency

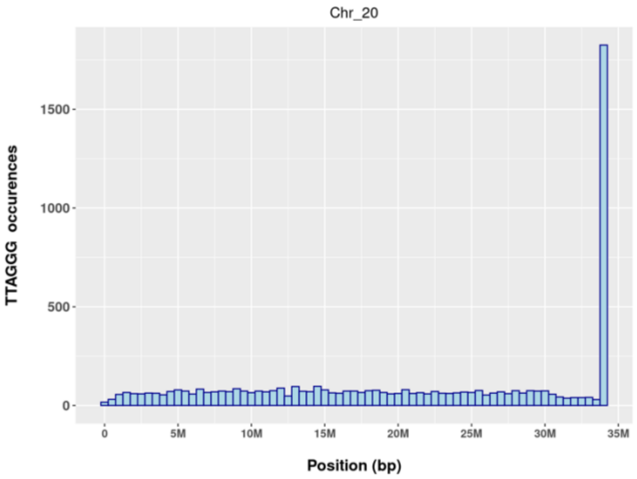

Density of pattern occurrence frequency

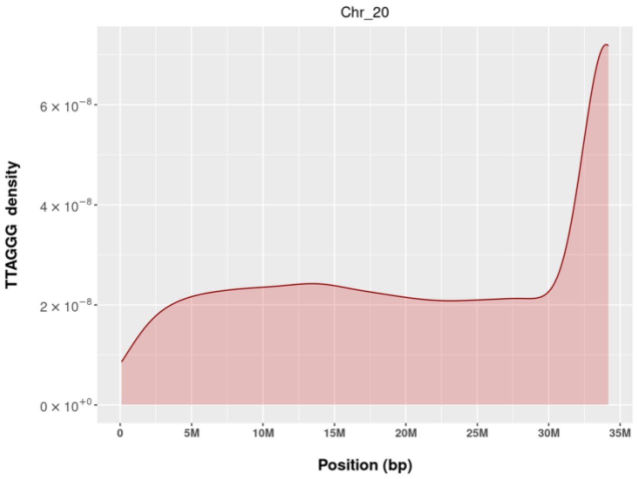

Chr_21

Histogram of pattern occurrence frequency

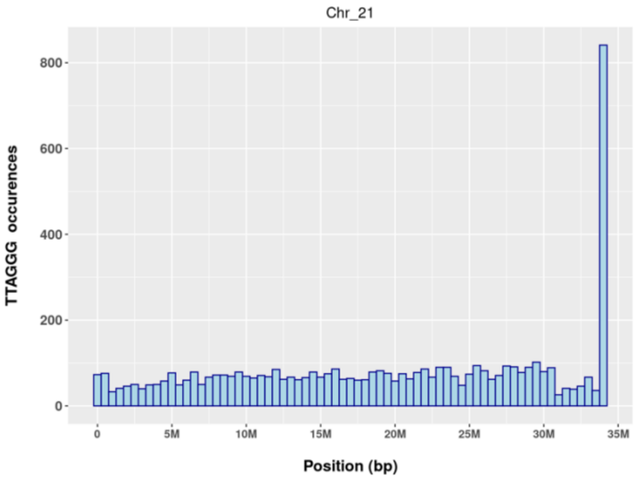

Density of pattern occurrence frequency

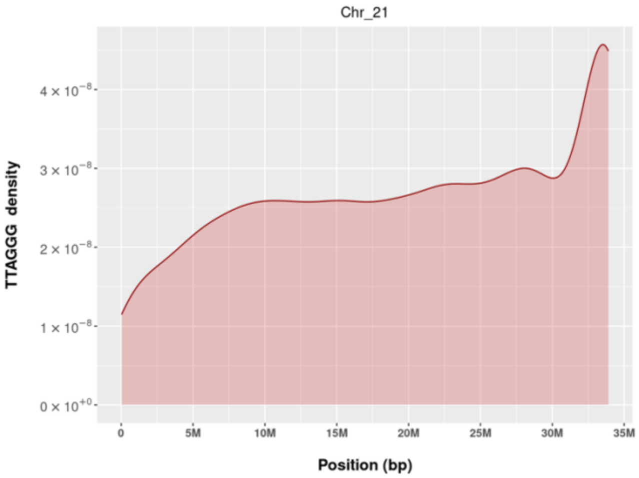

Chr_22

Histogram of pattern occurrence frequency

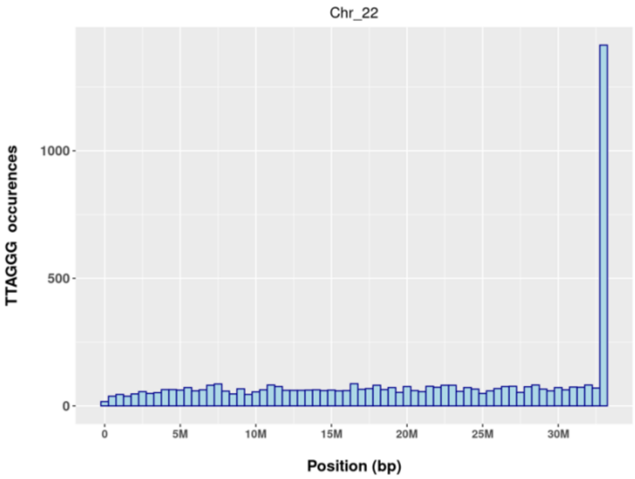

Density of pattern occurrence frequency

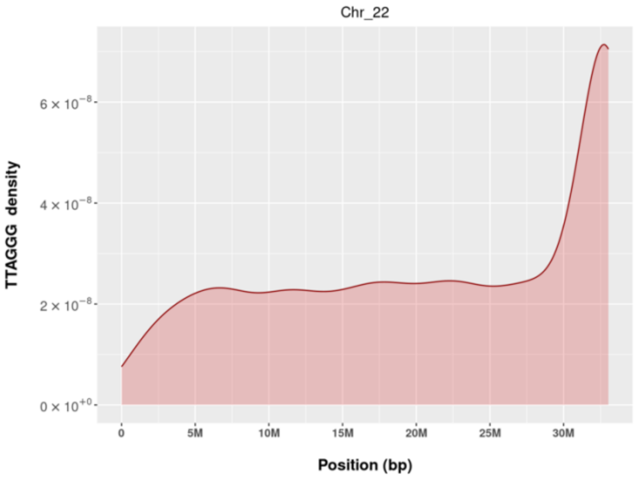

Chr_23

Histogram of pattern occurrence frequency

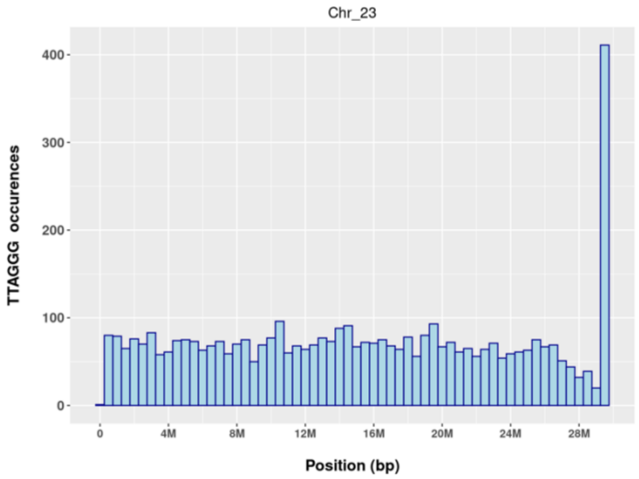

Density of pattern occurrence frequency

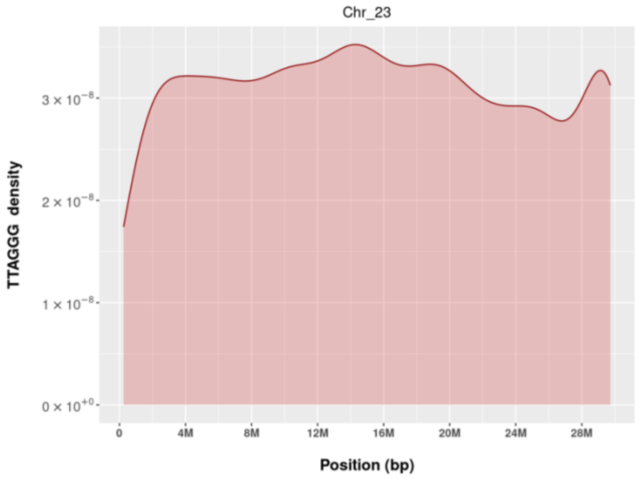

Chr_24

Histogram of pattern occurrence frequency

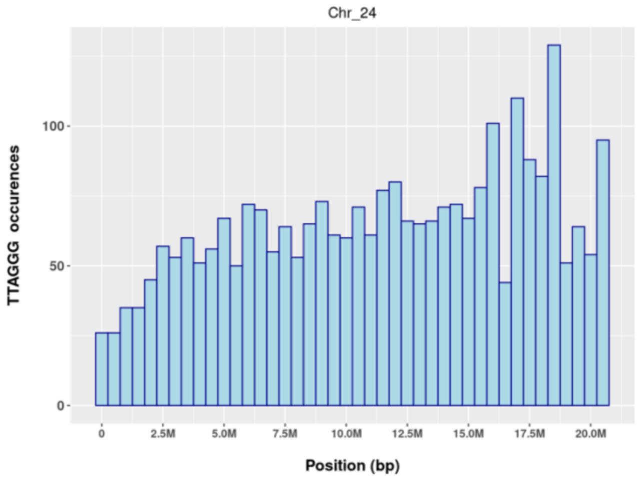

Density of pattern occurrence frequency

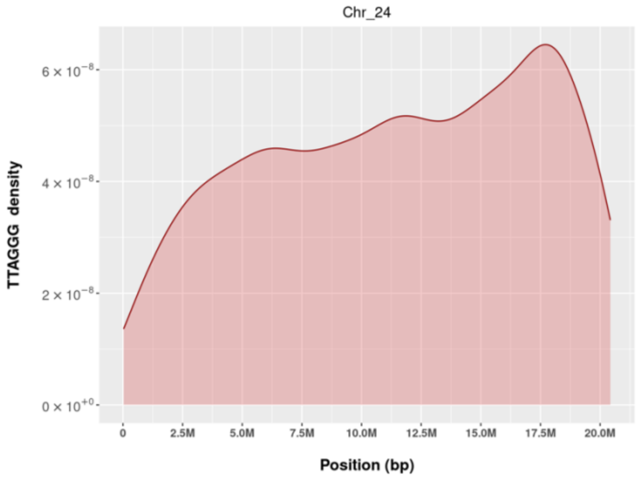

Pattern CCCTAA

Chr_01

Histogram of pattern occurrence frequency

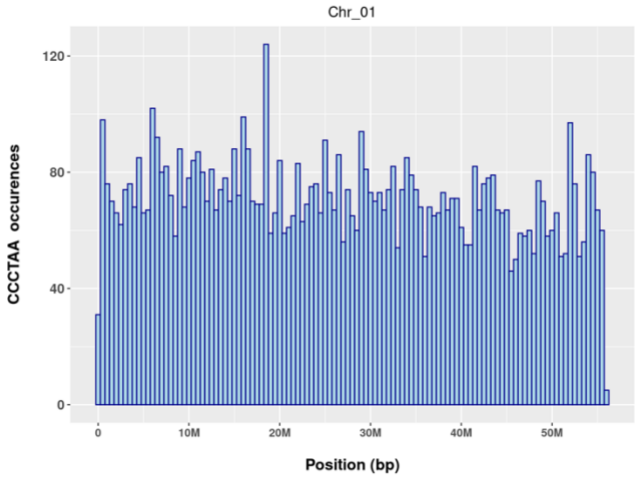

Density of pattern occurrence frequency

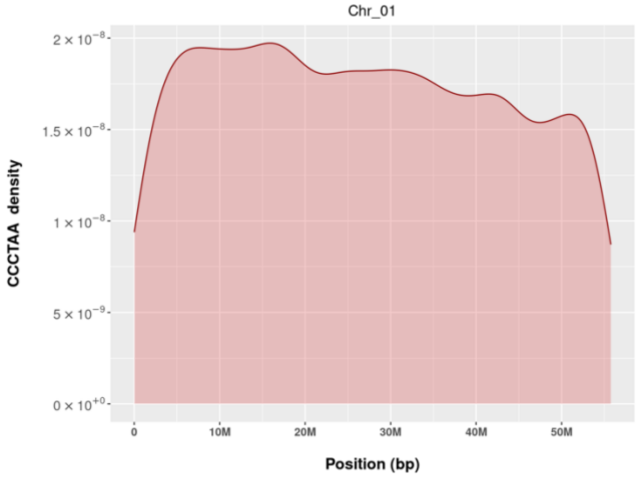

Chr_02

Histogram of pattern occurrence frequency

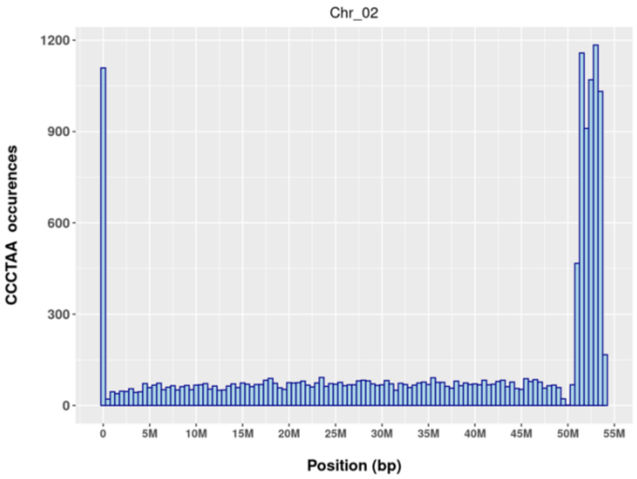

Density of pattern occurrence frequency

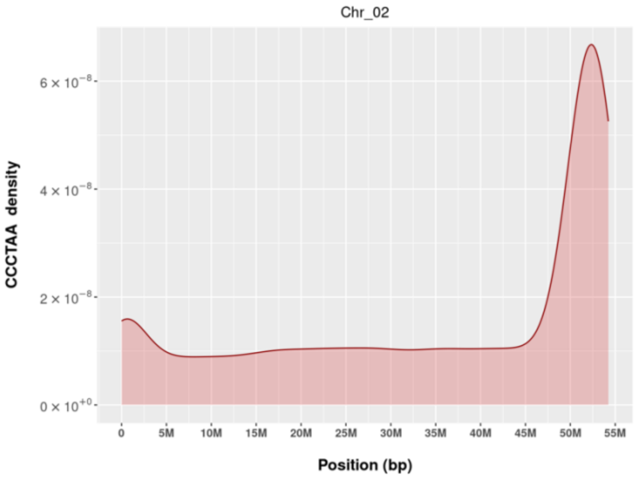

Chr_03

Histogram of pattern occurrence frequency

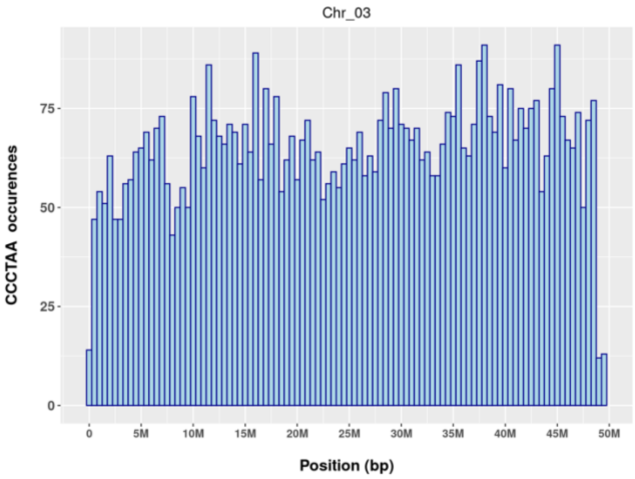

Density of pattern occurrence frequency

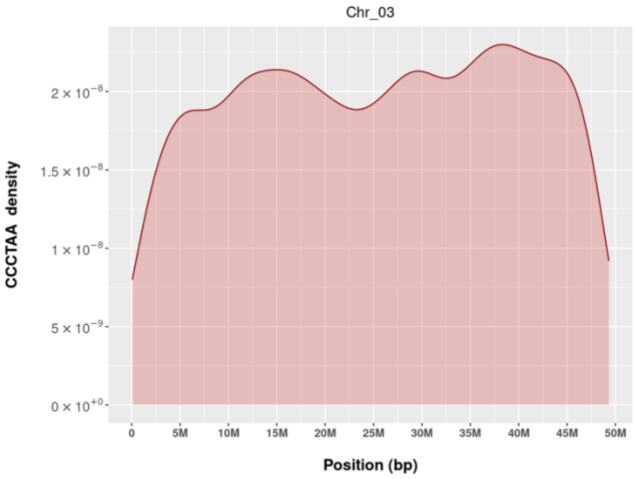

Chr_04

Histogram of pattern occurrence frequency

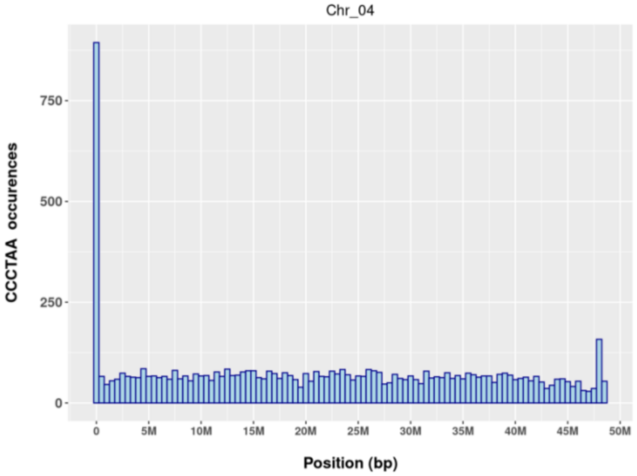

Density of pattern occurrence frequency

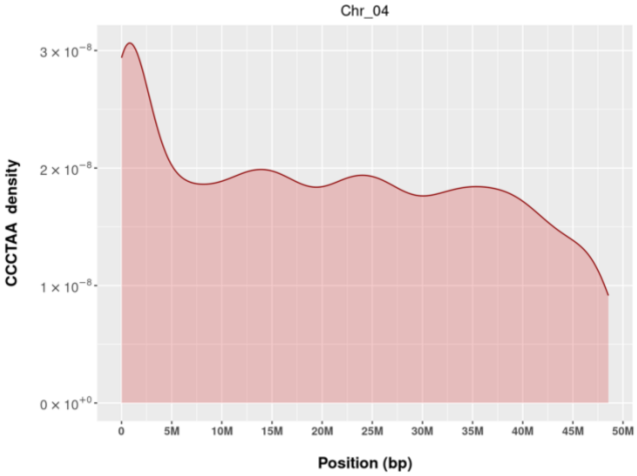

Chr_05

Histogram of pattern occurrence frequency

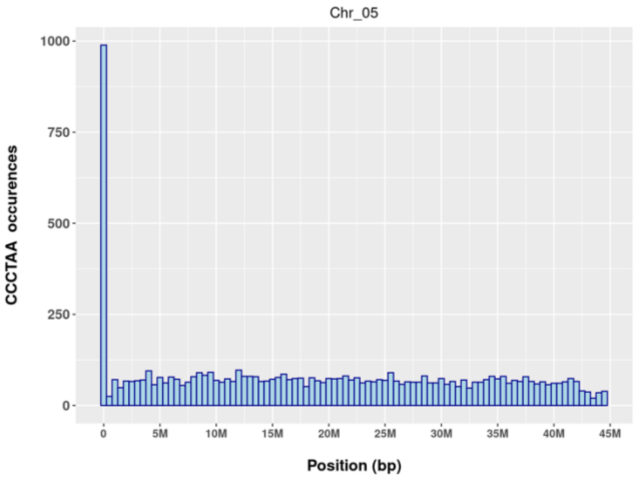

Density of pattern occurrence frequency

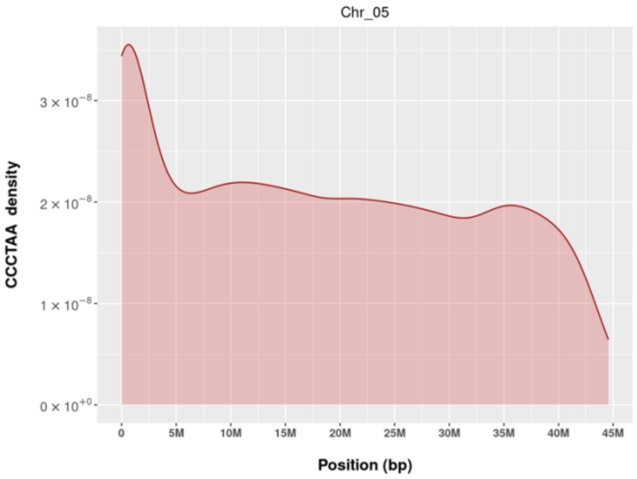

Chr_06

Histogram of pattern occurrence frequency

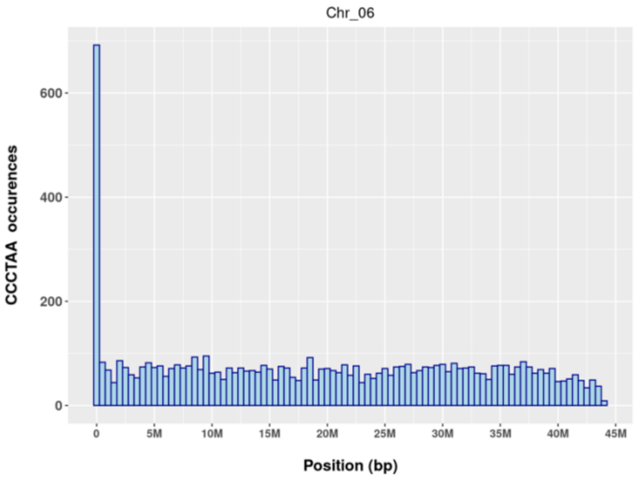

Density of pattern occurrence frequency

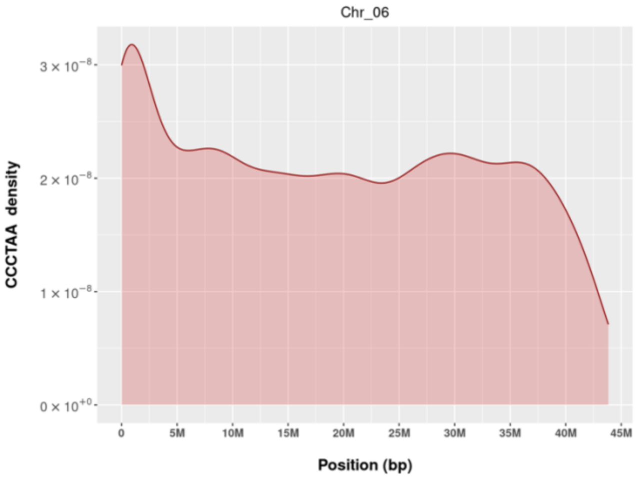

Chr_07

Histogram of pattern occurrence frequency

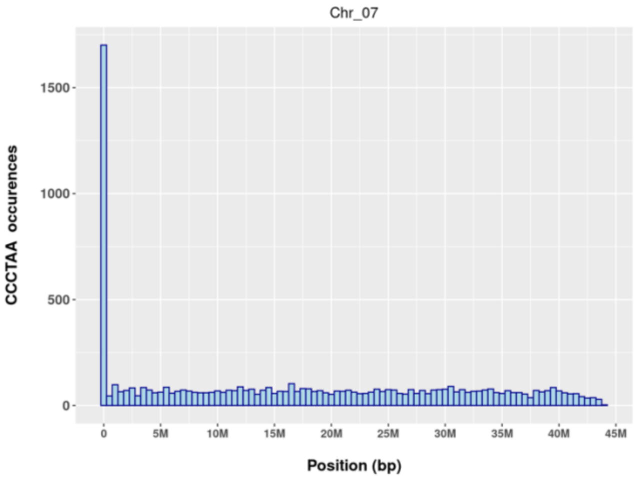

Density of pattern occurrence frequency

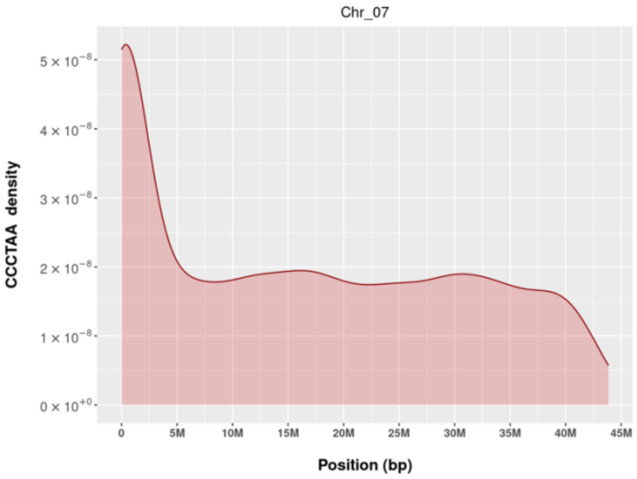

Chr_08

Histogram of pattern occurrence frequency

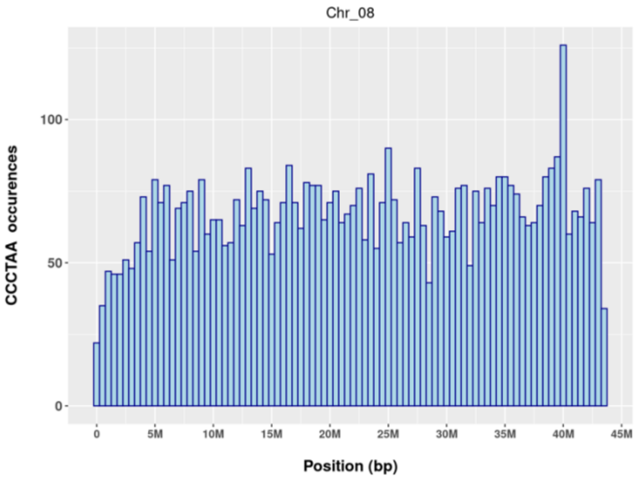

Density of pattern occurrence frequency

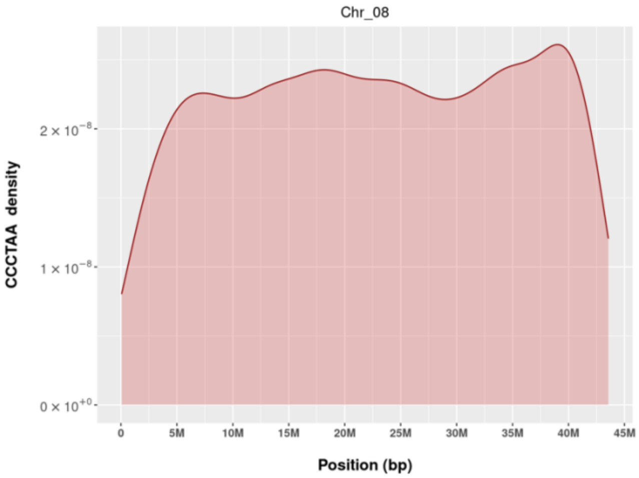

Chr_09

Histogram of pattern occurrence frequency

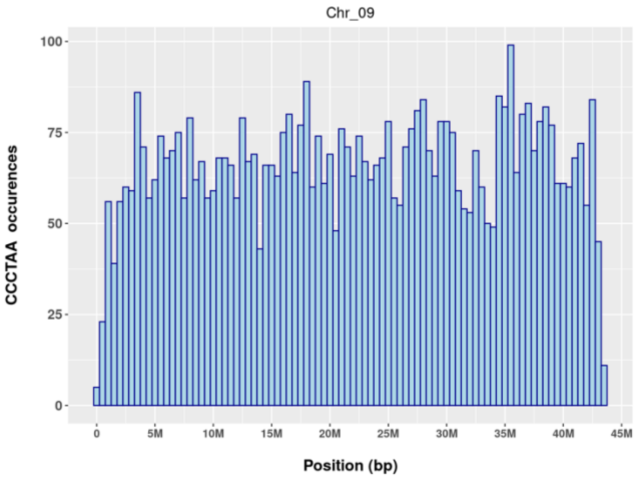

Density of pattern occurrence frequency

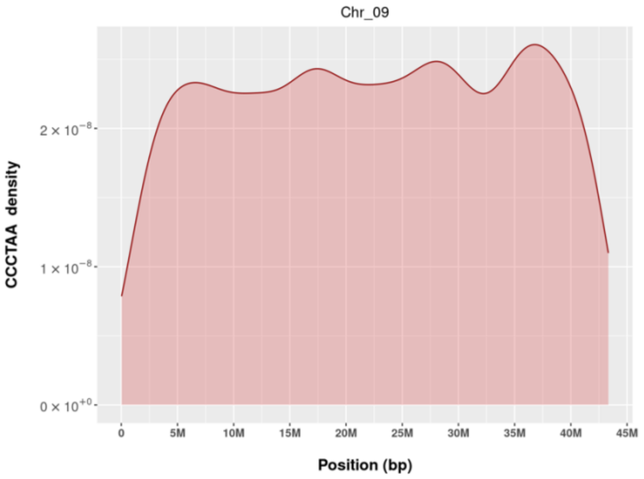

Chr_10

Histogram of pattern occurrence frequency

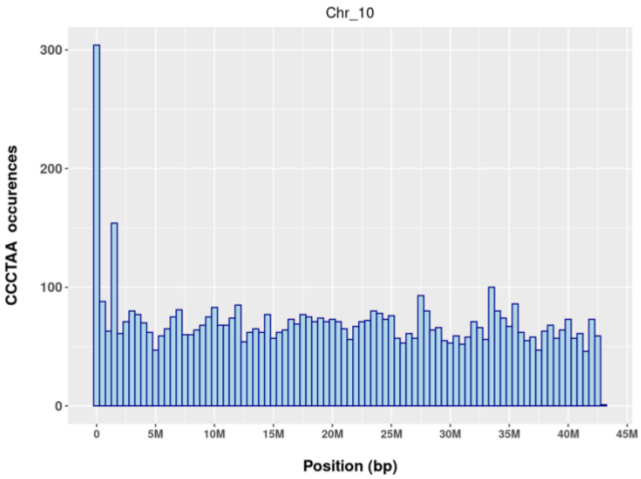

Density of pattern occurrence frequency

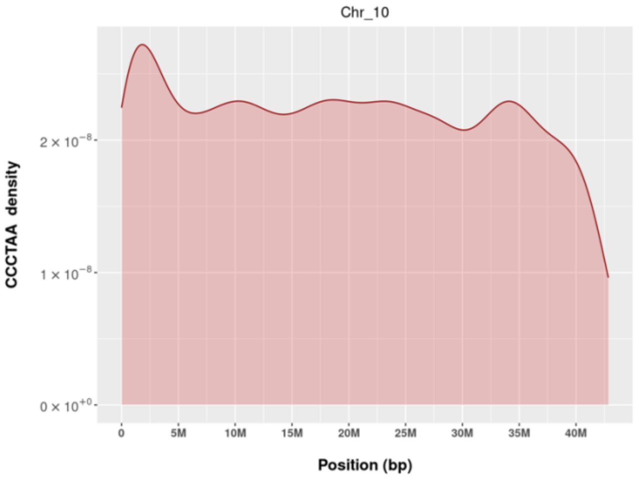

Chr_11

Histogram of pattern occurrence frequency

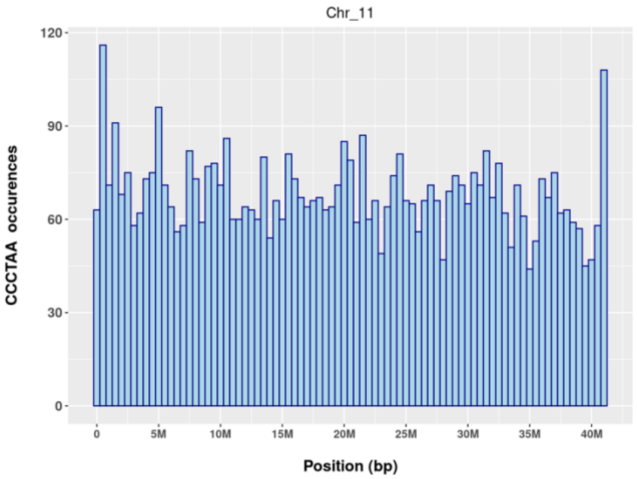

Density of pattern occurrence frequency

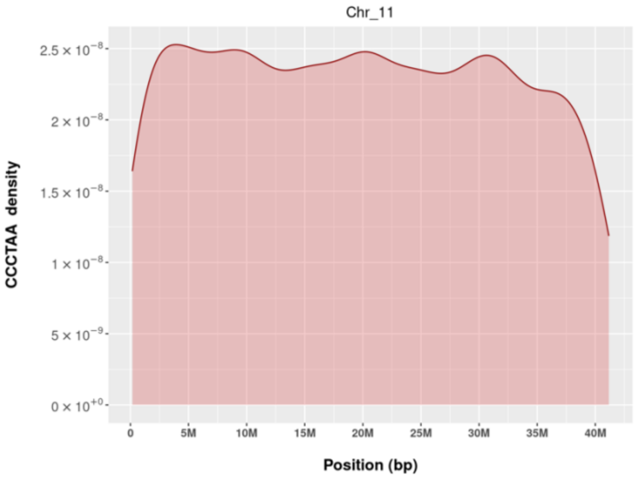

Chr_12

Histogram of pattern occurrence frequency

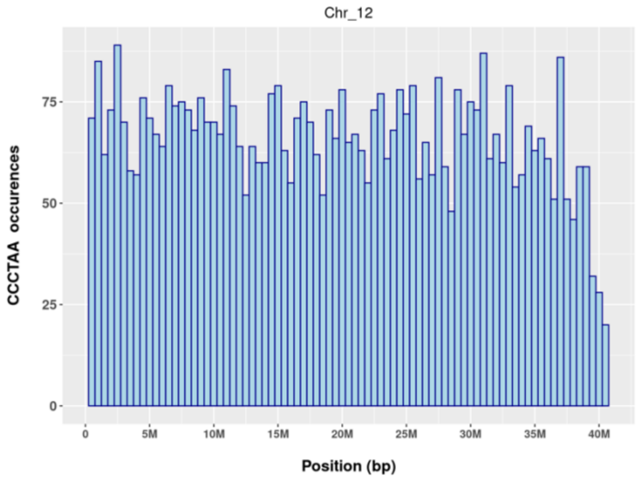

Density of pattern occurrence frequency

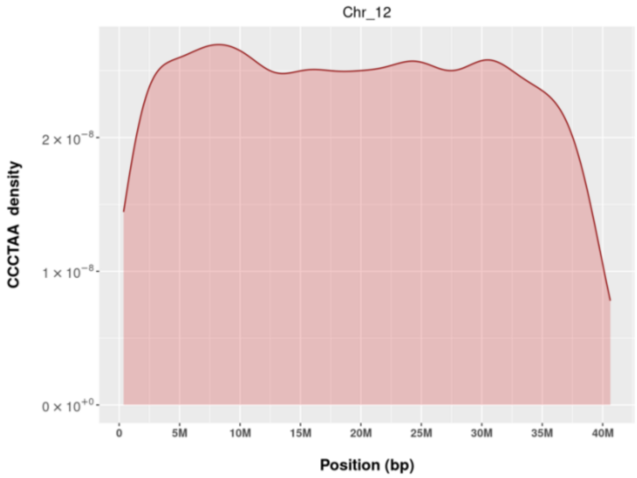

Chr_13

Histogram of pattern occurrence frequency

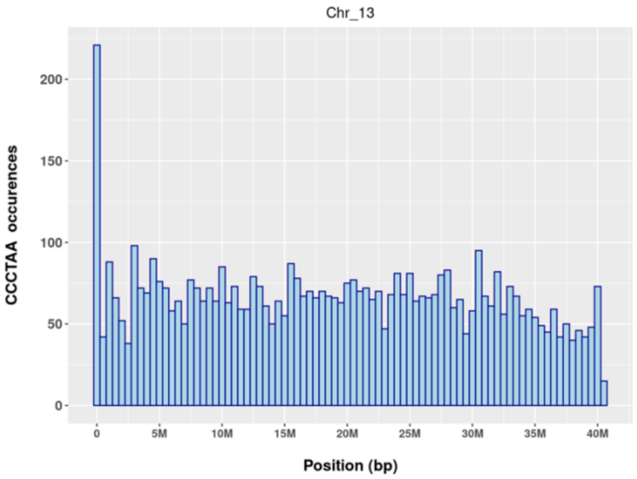

Density of pattern occurrence frequency

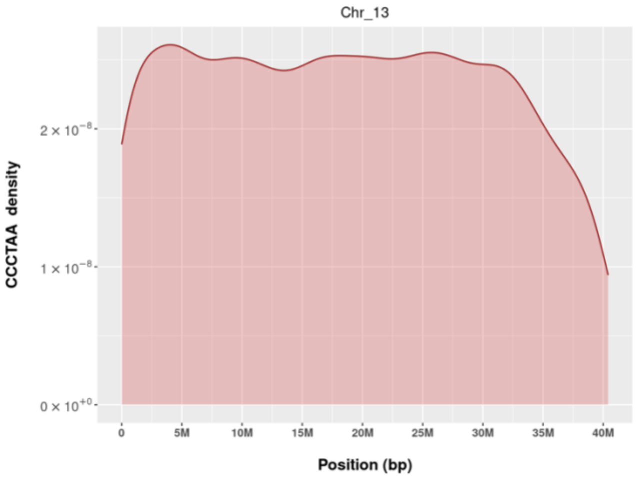

Chr_14

Histogram of pattern occurrence frequency

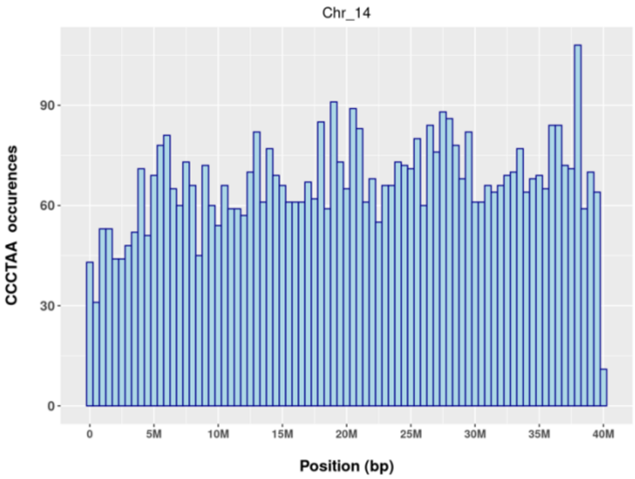

Density of pattern occurrence frequency

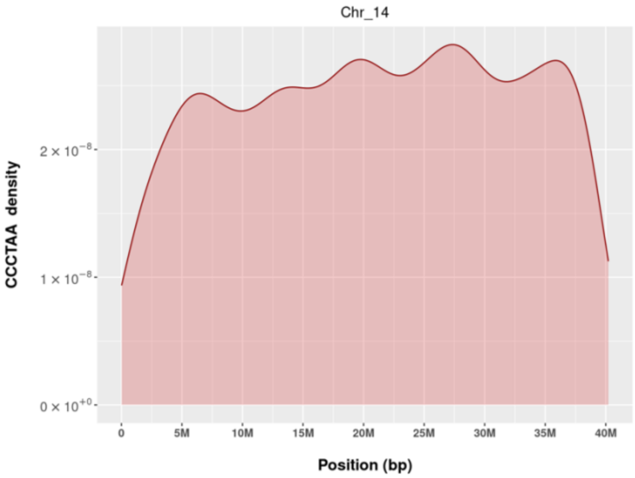

Chr_15

Histogram of pattern occurrence frequency

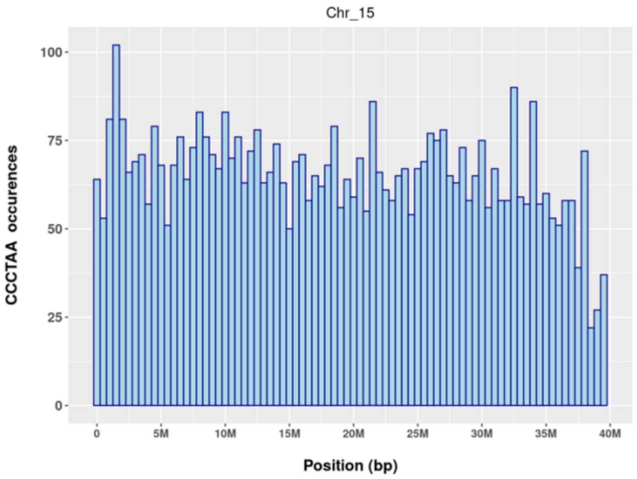

Density of pattern occurrence frequency

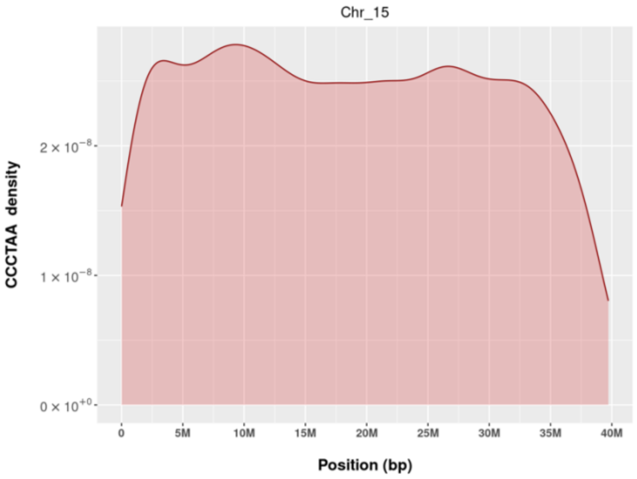

Chr_16

Histogram of pattern occurrence frequency

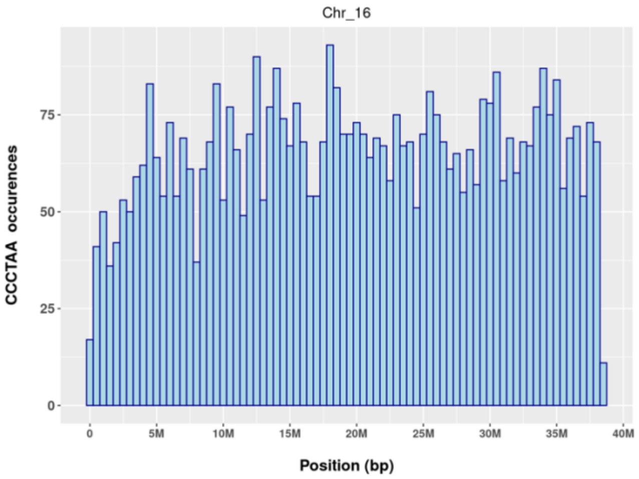

Density of pattern occurrence frequency

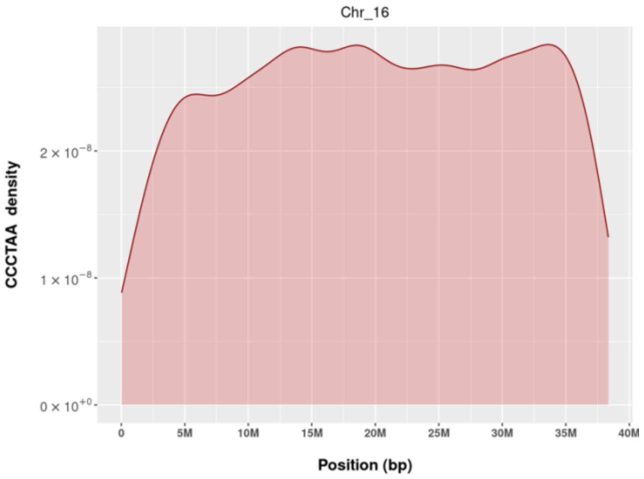

Chr_17

Histogram of pattern occurrence frequency

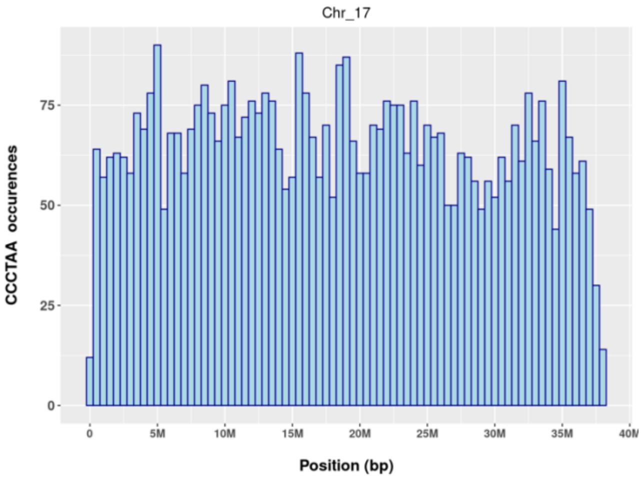

Density of pattern occurrence frequency

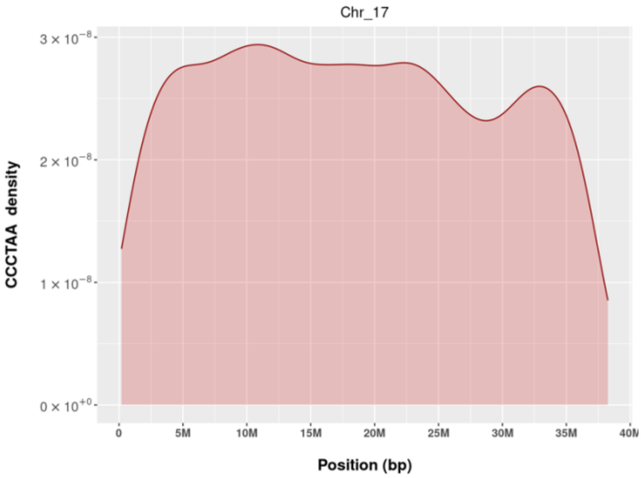

Chr_18

Histogram of pattern occurrence frequency

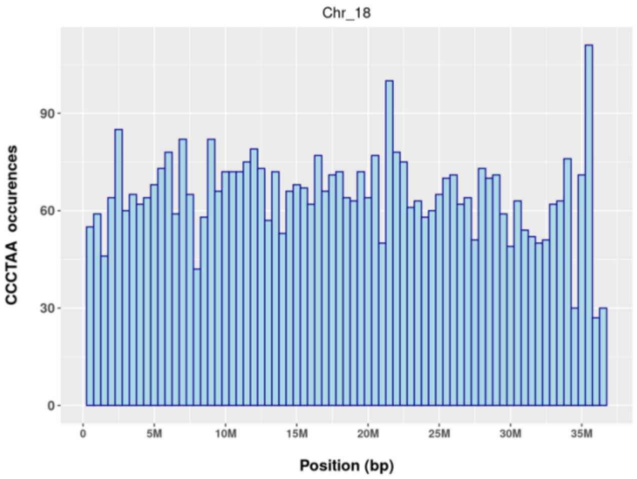

Density of pattern occurrence frequency

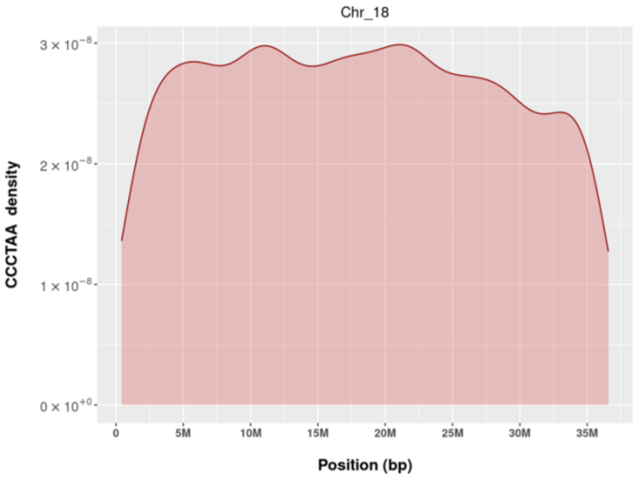

Chr_19

Histogram of pattern occurrence frequency

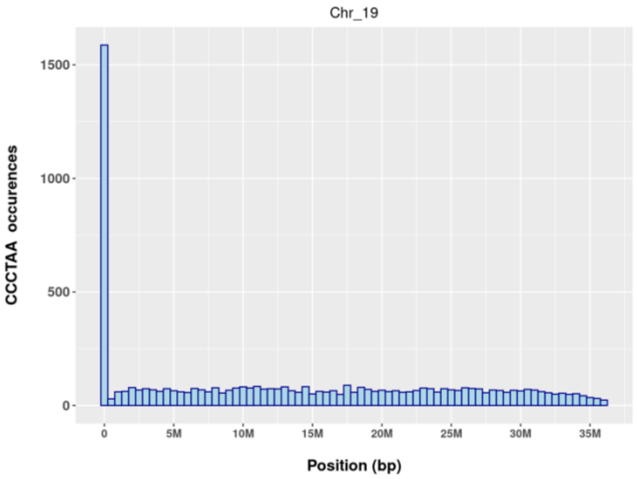

Density of pattern occurrence frequency

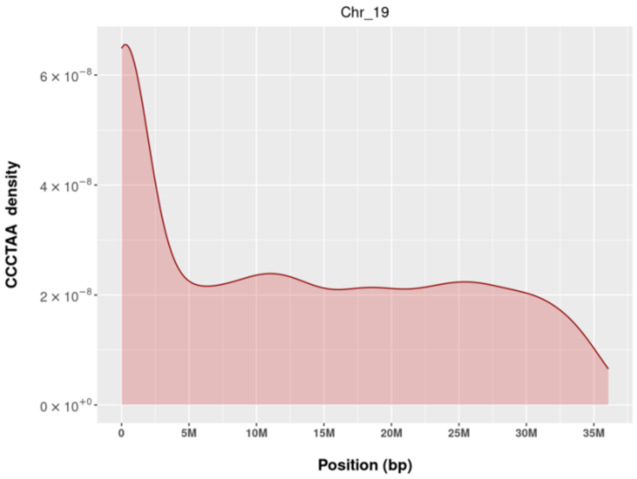

Chr_20

Histogram of pattern occurrence frequency

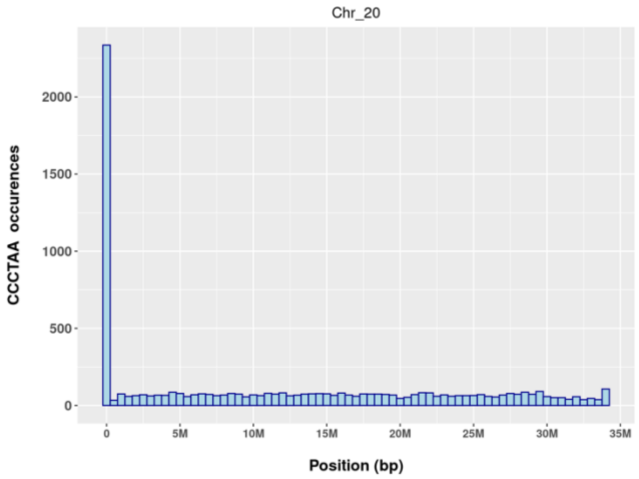

Density of pattern occurrence frequency

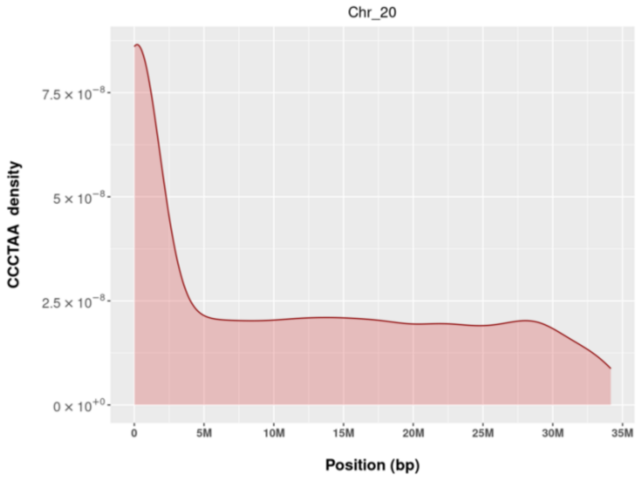

Chr_21

Histogram of pattern occurrence frequency

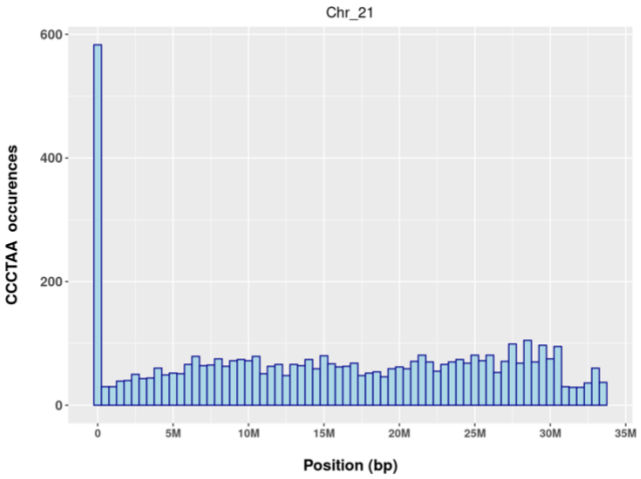

Density of pattern occurrence frequency

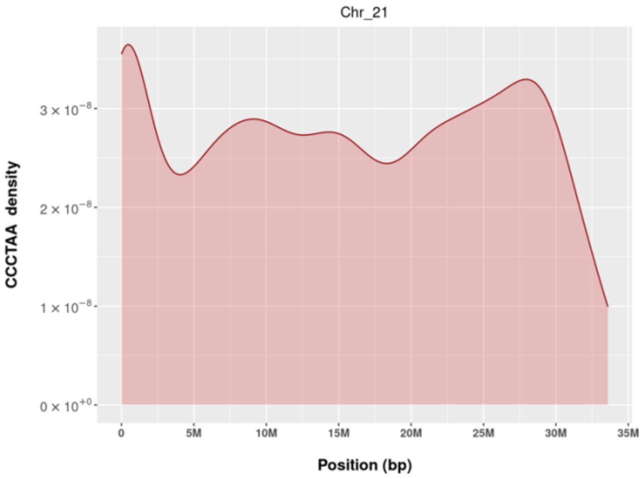

Chr_22

Histogram of pattern occurrence frequency

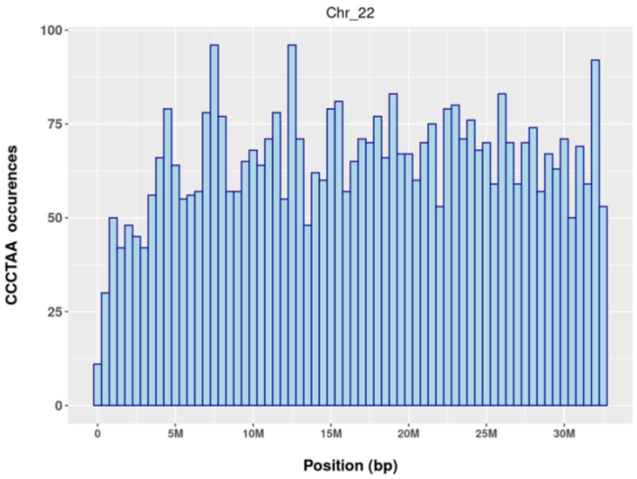

Density of pattern occurrence frequency

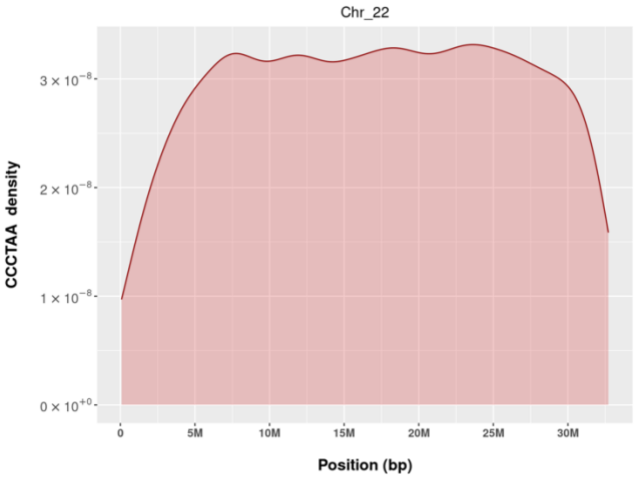

Chr_23

Histogram of pattern occurrence frequency

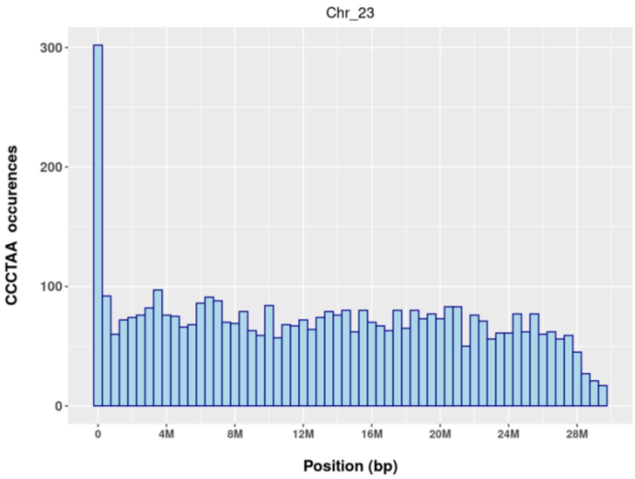

Density of pattern occurrence frequency

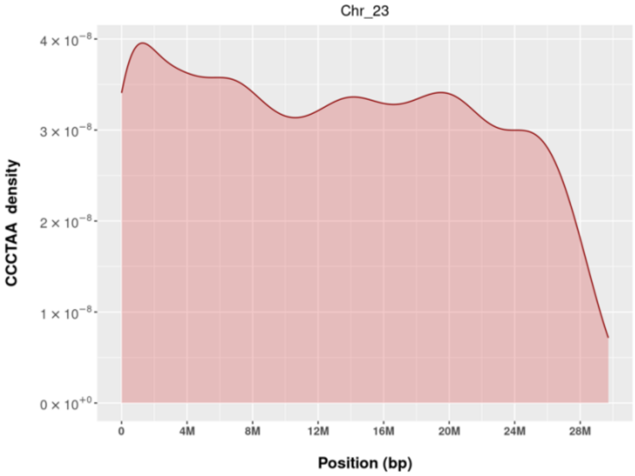

Chr_24

Histogram of pattern occurrence frequency

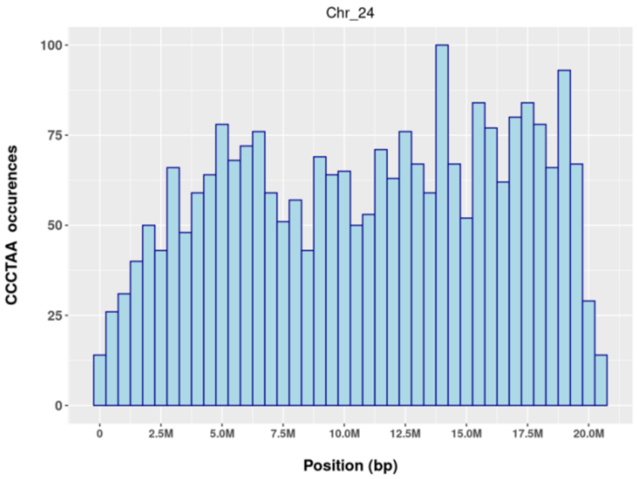

Density of pattern occurrence frequency

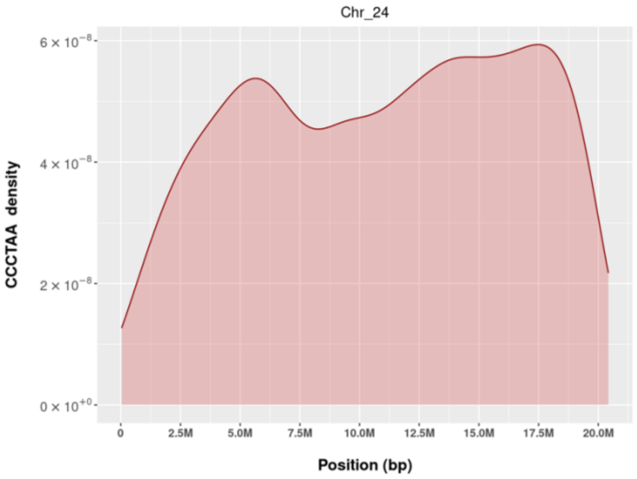

Supplement: Supplementary file 8 — Figure S2 [file 41597_2025_5573_MOESM8_ESM.pdf]
