# Supplementary material for: Chromosome-level genome assembly of the Vermilion Snapper (Rhomboplites aurorubens)
Source: Sci Data. 2025 Jul 23;12:1281. doi: 10.1038/s41597-025-05573-w (PMC12287329; doi:10.1038/s41597-025-05573-w)
Supplement: Supplementary file 9 — Figure S3 [file 41597_2025_5573_MOESM9_ESM.pdf]

Report

	vsnapper_v1_hifiasm	vsnapper_v1_3ddna	vsnapper_v1_endhic_100k	vsnapper_v1_endhic_150k
# contigs (>= 0 bp)	143	533	135	136
# contigs (>= 1000 bp)	143	533	135	136
# contigs (>= 5000 bp)	143	519	135	136
# contigs (>= 10000 bp)	143	512	135	136
# contigs (>= 25000 bp)	122	439	114	115
# contigs (>= 50000 bp)	66	167	58	59
Total length (>= 0 bp)	987478130	989292630	987486130	987485130
Total length (>= 1000 bp)	987478130	989292630	987486130	987485130
Total length (>= 5000 bp)	987478130	989260856	987486130	987485130
Total length (>= 10000 bp)	987478130	989207251	987486130	987485130
Total length (>= 25000 bp)	987015143	987664901	987023143	987022143
Total length (>= 50000 bp)	985023938	980011534	985031938	985030938
# contigs	143	533	135	136
Largest contig	52366744	41077325	56173364	82977889
Total length	987478130	989292630	987486130	987485130
GC (%)	40.49	40.49	40.49	40.49
N50	41288585	34028350	41288585	42864469
N90	32587949	5102896	33900093	33900093
auN	39818447.4	29063128.1	41402880.7	44594099.7
L50	11	14	11	10
L90	22	34	21	21
# N's per 100 kbp	0.00	183.41	0.81	0.71

All statistics are based on contigs of size >= 500 bp, unless otherwise noted
(e.g., "# contigs (>= 0 bp)" and "Total length (>= 0 bp)" include all contigs).

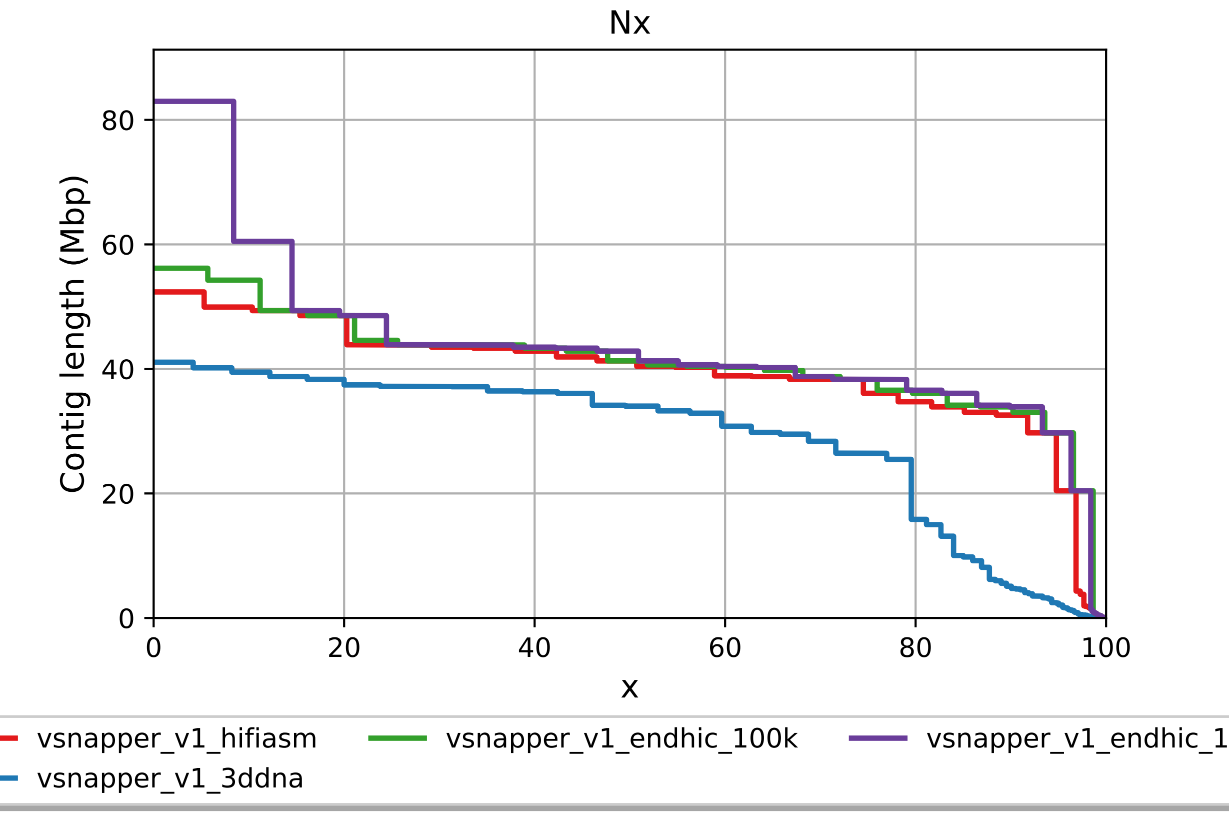
Nx
Contig length (Mbp)
0
20
40
60
80
0
20
40
60
80
100
x
vsnapper_v1_hifiasm
vsnapper_v1_endhic_100k
vsnapper_v1_endhic_1
vsnapper_v1_3ddna

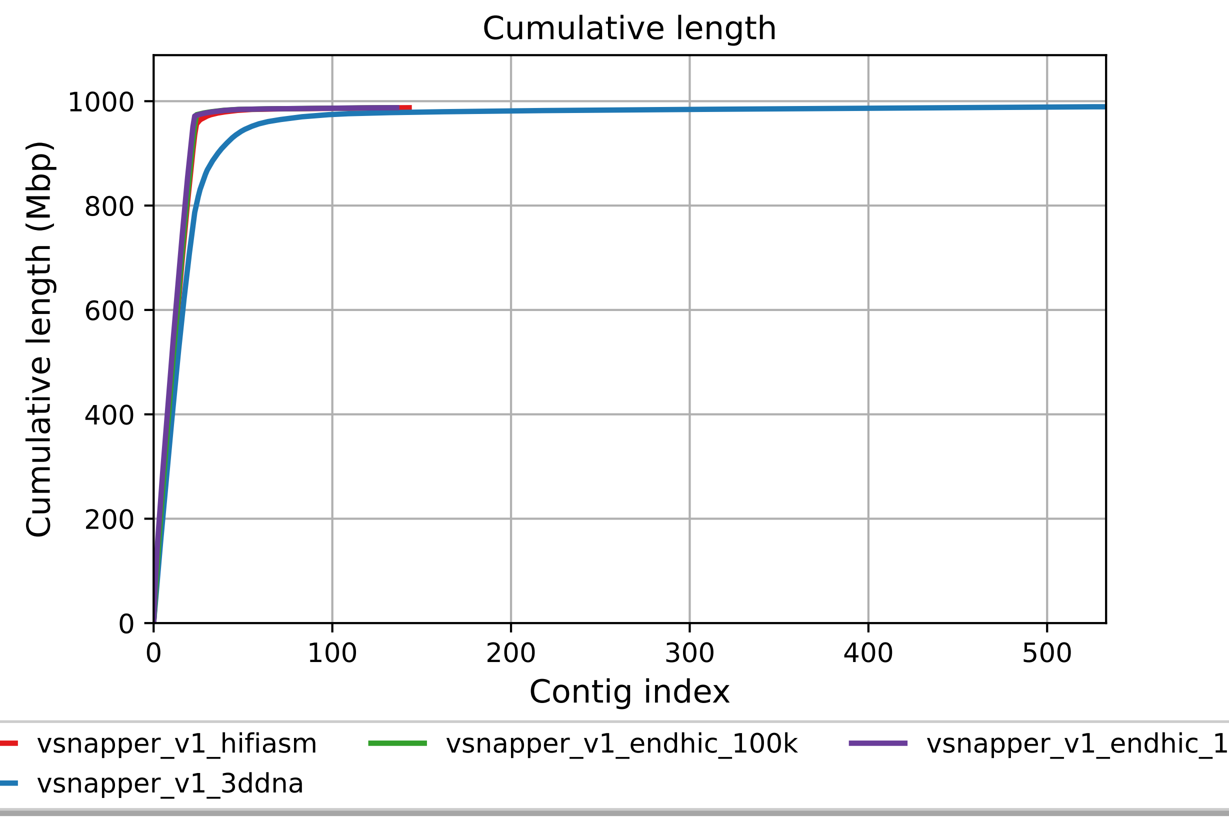

Cumulative length
Cumulative length (Mbp)
0
200
400
600
800
1000
0
100
200
300
400
500
Contig index
vsnapper_v1_hifiasm
vsnapper_v1_endhic_100k
vsnapper_v1_endhic_1
vsnapper_v1_3ddna

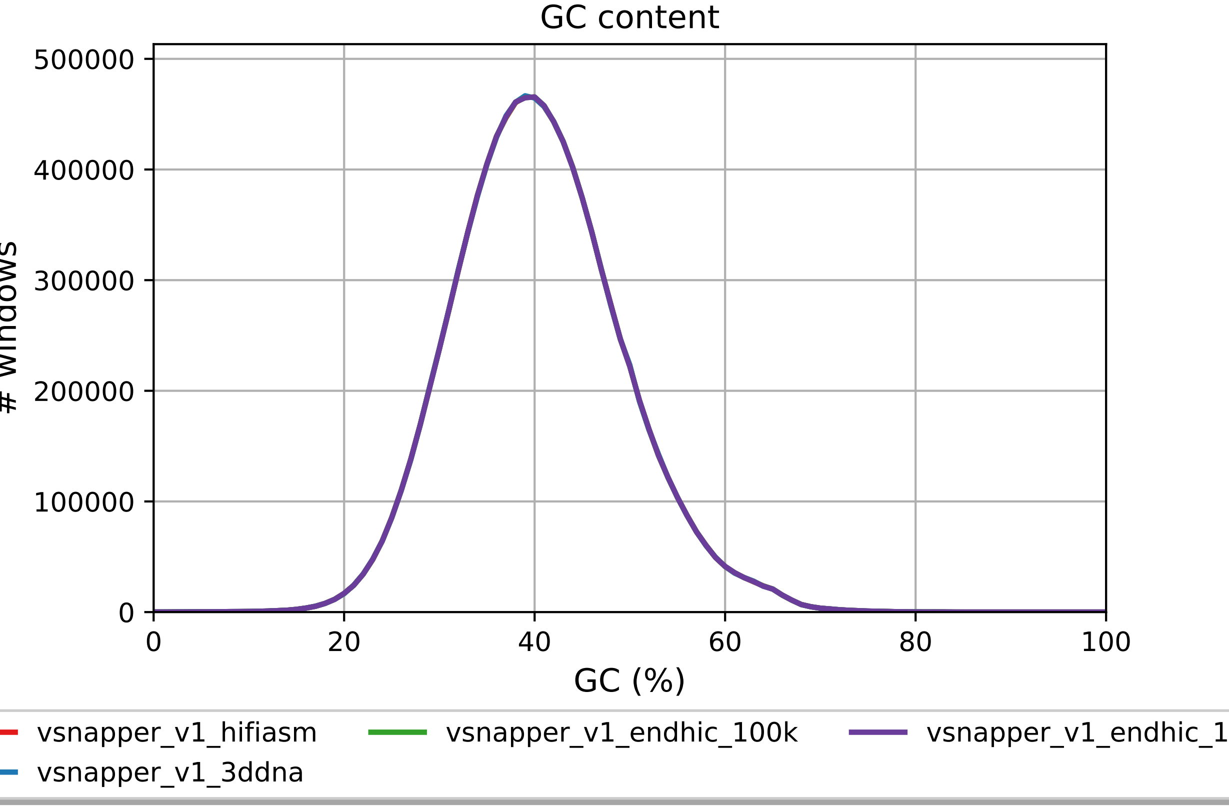
GC content
500000
400000
300000
200000
100000
0
windows
0
20
40
60
80
100
GC (%)
vsnapper_v1_hifiasm
vsnapper_v1_endhic_100k
vsnapper_v1_endhic_1
vsnapper_v1_3ddna

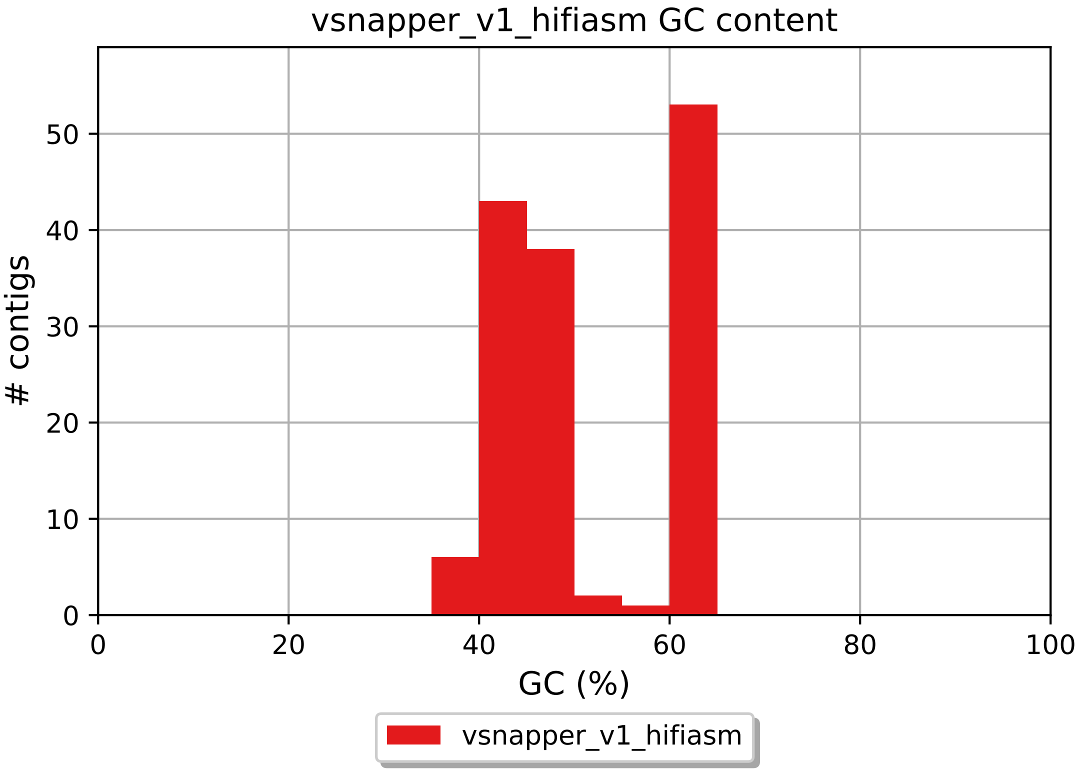
vsnapper_v1_hifiasm GC content
contigs
0
10
20
30
40
50
0
20
40
60
80
100
GC (%)
vsnapper_v1_hifiasm

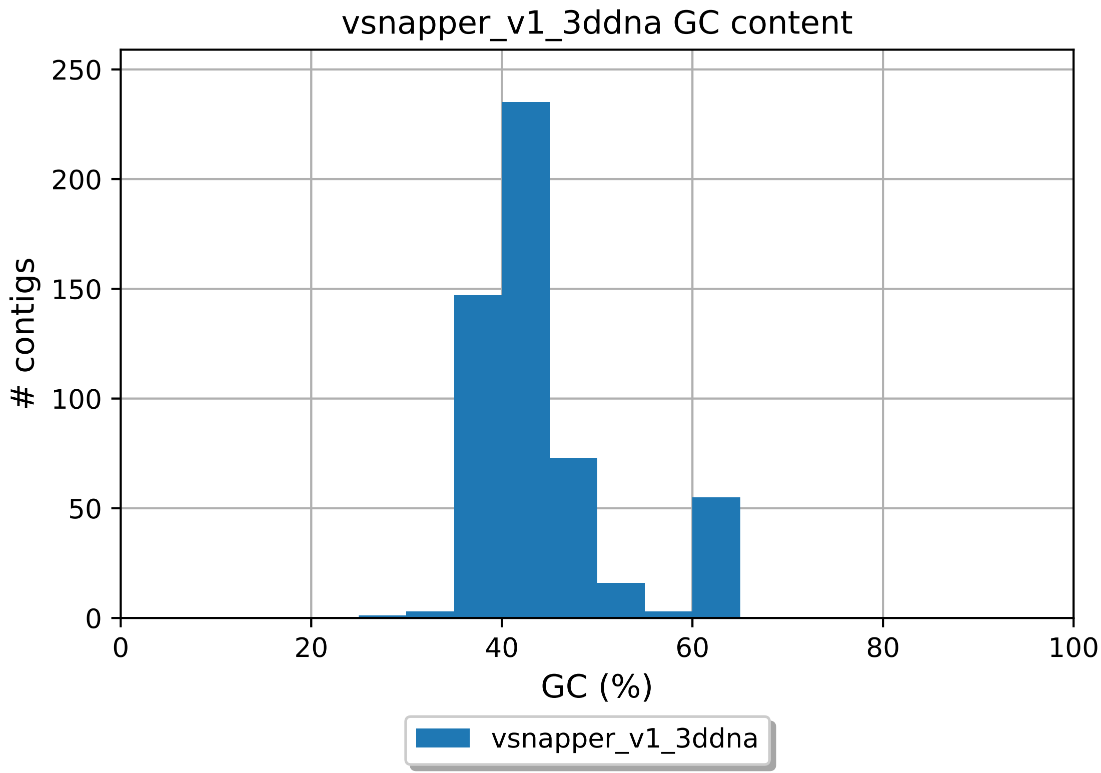

vsnapper_v1_3ddna GC content
contigs
GC (%)
0
50
100
150
200
250
0
20
40
60
80
100
vsnapper_v1_3ddna

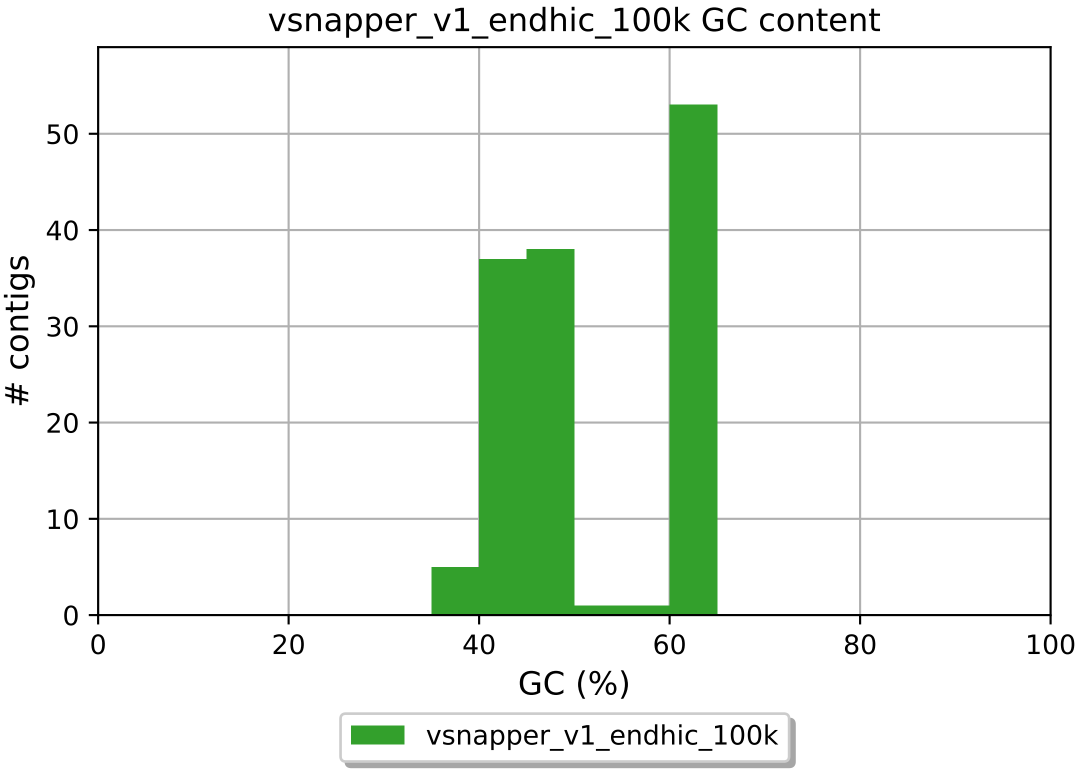

vsnapper_v1_endhic_100k GC content
contigs
0
10
20
30
40
50
0
20
40
60
80
100
GC (%)
vsnapper_v1_endhic_100k

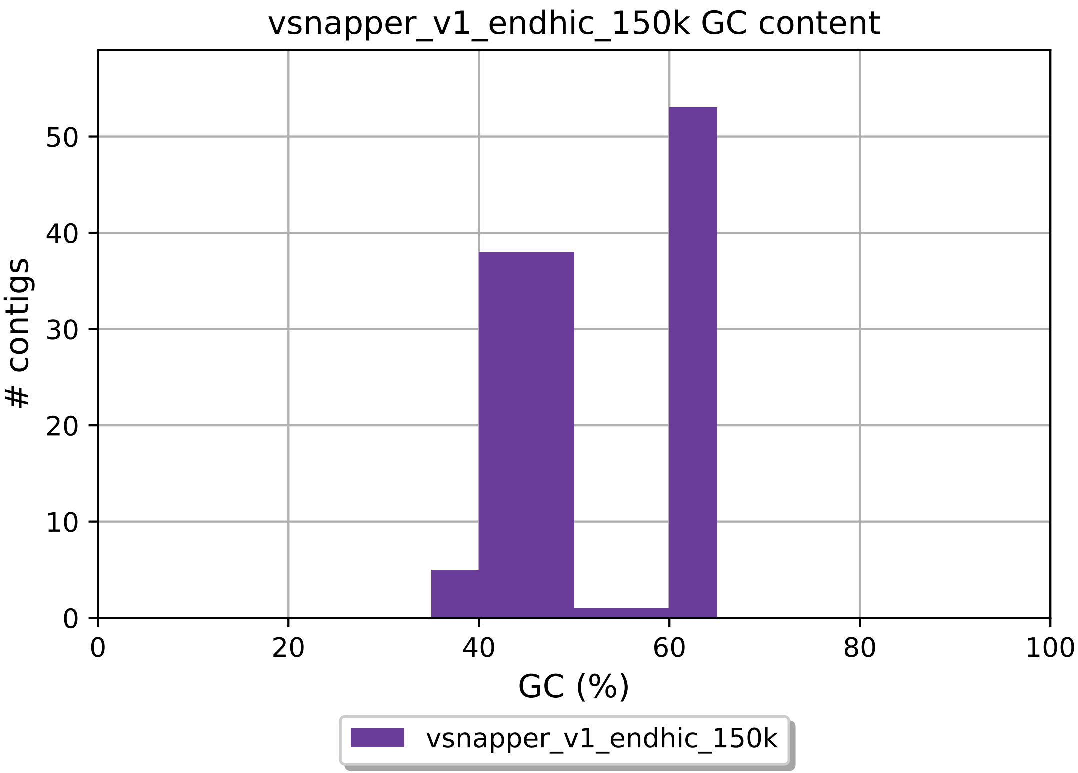
vsnapper_v1_endhic_150k GC content
contigs
0
10
20
30
40
50
0
20
40
60
80
100
GC (%)
vsnapper_v1_endhic_150k
